# Comparative phytochemistry of flavaglines (= rocaglamides), a group of highly bioactive flavolignans from *Aglaia* species (Meliaceae)

**DOI:** 10.1007/s11101-021-09761-5

**Published:** 2021-06-04

**Authors:** Harald Greger

**Affiliations:** Chemodiversity Research Group, Faculty of Life Sciences, University of Vienna, Rennweg 14, 1030 Wien, Austria

**Keywords:** *Aglaia*, Meliaceae, Flavaglines, Rocaglamides, Rocaglates, Cyclopentabenzofurans, Cyclopentabenzopyrans, Benzoxepines, Structural relationships, Chemotaxonomy, Biological activities, Translational repression, Insecticidal, Antiprotozoal, Antifungal, Anticancer, Antiviral, Anti-inflammatory

## Abstract

Flavaglines are formed by cycloaddition of a flavonoid nucleus with a cinnamic acid moiety representing a typical chemical character of the genus *Aglaia* of the family Meliaceae. Based on biosynthetic considerations 148 derivatives are grouped together into three skeletal types representing 77 cyclopenta[*b*]benzofurans, 61 cyclopenta[*bc*]benzopyrans, and 10 benzo[*b*]oxepines. Apart from different hydroxy, methoxy, and methylenedioxy groups of the aromatic rings, important structural variation is created by different substitutions and stereochemistries of the central cyclopentane ring. Putrescine-derived bisamides constitute important building blocks occurring as cyclic 2-aminopyrrolidines or in an open-chained form, and are involved in the formation of pyrimidinone flavaglines. Regarding the central role of cinnamic acid in the formation of the basic skeleton, rocagloic acid represents a biosynthetic precursor from which aglafoline- and rocaglamide-type cyclopentabenzofurans can be derived, while those of the rocaglaol-type are the result of decarboxylation. Broad-based comparison revealed characteristic substitution trends which contribute as chemical markers to natural delimitation and grouping of taxonomically problematic *Aglaia* species. A wide variety of biological activities ranges from insecticidal, antifungal, antiprotozoal, and anti-inflammatory properties, especially to pronounced anticancer and antiviral activities. The high insecticidal activity of flavaglines is comparable with that of the well-known natural insecticide azadirachtin. Comparative feeding experiments informed about structure–activity relationships and exhibited different substitutions of the cyclopentane ring essential for insecticidal activity. Parallel studies on the antiproliferative activity of flavaglines in various tumor cell lines revealed similar structural prerequisites that let expect corresponding molecular mechanisms. An important structural modification with very high cytotoxic potency was found in the benzofuran silvestrol characterized by an unusual dioxanyloxy subunit. It possessed comparable cytotoxicity to that of the natural anticancer compounds paclitaxel (Taxol®) and camptothecin without effecting normal cells. The primary effect was the inhibition of protein synthesis by binding to the translation initiation factor eIF4A, an ATP-dependent DEAD-box RNA helicase. Flavaglines were also shown to bind to prohibitins (PHB) responsible for regulation of important signaling pathways, and to inhibit the transcriptional factor HSF1 deeply involved in metabolic programming, survival, and proliferation of cancer cells. Flavaglines were shown to be not only promising anticancer agents but gained now also high expectations as agents against emerging RNA viruses like SARS-CoV-2. Targeting the helicase eIF4A with flavaglines was recently described as pan-viral strategy for minimizing the impact of future RNA virus pandemics.

## Introduction

In the course of our broad-based bioassay-guided screening for naturally occurring insecticides and fungicides in tropical rainforests we found exceptionally high insecticidal activities in some *Aglaia* species of the family Meliaceae. In accordance with previous findings (Satasook et al. 1992; Janprasert et al. 1993; Ishibashi et al. 1993; Ewete et al. 1996; Nugroho et al. 1997a, b; Güssregen et al. 1997) the activities could be attributed to a group of cyclopenta[*b*]benzofurans (Brader et al. 1998; Bacher et al. 1999). Some derivatives were shown to have also antifungal properties, displaying high activity particularly against *Pyricularia grisea*, the causative fungus of rice blast disease (Engelmeier et al. 2000). Continuative studies, especially by Proksch and collaborators, revealed further insecticidal derivatives which all were characterized by a benzofuran basic skeleton (Hiort et al. 1999; Chaidir et al. 1999, 2001; Molleyres et al. 1999; Nugroho et al. 1999; Schneider et al. 2000; Dreyer et al. 2001; Greger et al. 2001; Bringmann et al. 2003; Koul et al. 2004, 2005; Duong 2005; Hall et al. 2017).

The formation of cyclopenta[*b*]benzofurans was shown to represent a characteristic chemical feature of the genus *Aglaia* not detected so far in any other genus of the Meliaceae. The first derivative of this group, rocaglamide (**25**), exhibited significant antileukemic activity and was isolated from *Aglaia rimosa* (Blanco) Merrill (= *A. elliptifolia* Merrill) collected in Taiwan (King et al. 1982). As a consequence, apart from ongoing screenings for insecticidal properties, there has also been a great parallel interest in that class of compounds as potential anticancer agents (Ohse et al. 1996; Wu et al. 1997; Cui et al. 1997; Lee et al. 1998; Bohnenstengel et al. 1999a,b; Wang and Duh 2001; Hausott et al. 2004; Mi et al. 2006a; Zhu et al. 2007, 2009; Cencic et al. 2009; Ribeiro et al. 2012; Basmadjian et al. 2013). Moreover, anti-inflammatory (Baumann et al. 2002; Proksch et al. 2005; Fahrig et al. 2005; Salim et al. 2007b), antiprotozoal (Phongmaykin et al. 2011; Astelbauer et al. 2011, 2012; Langlais et al. 2018; Drinić et al. 2019), and more recently, antiviral activities (Biedenkopf et al. 2017; Müller et al. 2018, 2020, 2021; Todt et al. 2018; Glitscher et al. 2018; Elgner et al. 2018; Henss et al. 2018; Schulz et al. 2021; Taroncher-Oldenburg et al. 2021) were also reported which greatly stimulated further research in this class of compounds.

The discovery of structurally related derivatives with a cyclopenta[*bc*]benzopyran skeleton in the leaves of *A. argentea* Blume and benzo[*b*]oxepines in the bark of *A. forbesii* King, coexisting with flavonoids and bisamides, suggested a common biogenetic origin (Dumontet et al. 1996). In two subsequent reports a biosynthetic pathway was proposed independently, suggesting the formation of a flavolignan basic skeleton created by cycloaddition between a flavonoid nucleus and a cinnamic acid moiety (Bacher et al. 1999; Nugroho et al. 1999). For the sake of convenience the benzofuran derivatives were previously designated as rocaglamides following the trivial name of the first derivative (Proksch et al. 2001). However, the benzofurans isolated from other *Aglaia* species, e.g. from *A. elliptica* Blume (Lee et al. 1998), *A. elaeagnoidea* (A. Juss.) Benth. (Brader et al. 1998) or *A. spectabilis* (Miq.) Jain & Bennet (Schneider et al. 2000), were shown to be devoid of nitrogen. Hence, the name rocaglamides as general denomination for this class of compounds appeared to be not appropriate and, instead, we have proposed their designation as flavaglines, regarding their restricted occurrence in the genus *Aglaia* and the incorporation of a flavonoid moiety as a central building block (Brader et al. 1998).

On the basis of our broad-based UV-HPLC comparison of 30 different *Aglaia* species^1^ it became apparent that benzofuran flavaglines are mainly accumulated in the stem bark and roots, while benzopyran flavaglines are dominating in the leaves, coexisting with bisamides, lignans, and flavonoids (Brem 2002). However, in some species no flavaglines could be detected in this preliminary survey, where only small quantities of plant material were used. In this case, mostly bisamides and/or lignans were shown to be the dominating compounds in the UV-HPLC-profiles, sometimes accumulated in all three plant parts, the leaves, stem bark, and roots (Greger et al. 2000, 2008; Bachratá 2008). The ecological impact of flavagline formation became apparent in parallel bioassays of crude extracts against the polyphagous pest insect *Spodoptera littoralis*, where pronouced insect toxicity was observed only in those species which showed an accumulation of benzofuran flavaglines (Greger et al. 2000, 2001, 2008; Brem 2002). Investigations within the distribution area of *Aglaia*, ranging from Sri Lanka to Fiji islands, did not show significant differences in the biogenetic capacity towards flavagline formation between species of the western part to those of the eastern part, across the Wallace's line (Greger et al. 2001; Muellner et al. 2005, 2009).

Regarding the results available so far flavaglines are unlikely to be developed into a commercial insecticide (Ebada et al. 2011; Hall et al. 2017), but their remarkable activity against cancer cell lines at nanomolar concentrations makes them extremly attractive as therapeutic agent candidates in cancer chemotherapy, even more, as they did not display major toxicity on normal cells (Hausott et al. 2004; Su et al. 2006; Zhu et al. 2007; Ribeiro et al. 2012; Pan et al. 2013; Callahan et al. 2014; Emhemmed et al. 2019). The primary effect of their antiproliferative activity was reported to be due to inhibition of protein synthesis (Ohse et al. 1996; Lee et al. 1998). It is now known that this effect is due to the binding of flavaglines to the translation initiation factor eIF4A, an ATP-dependent DEAD-box RNA helicase (Bordeleau et al. 2008; Cencic et al. 2009; Sadlish et al. 2013). Targeting the prohibitins-1 and 2, two evolutionarily conserved and ubiquitously expressed proteins controlling important cellular processes, was also discussed (Polier et al. 2012; Thuaud et al. 2013; Basmadjian et al. 2013, 2015). Further molecular mechanisms were summarized by Li-Weber (2015). The discovery of the exceptionally high activities of silvestrol (**14**), a benzofuran flavagline linked with an unusual 1,4-dioxanyloxy or “pseudosugar” substituent (Hwang et al. 2004), has greatly accelerated further pharmacological investigations, principally for the treatment of cancer (Kim et al. 2006b; Lucas et al. 2009; Aktas et al. 2011; Basmadjian et al. 2013; Pan et al. 2014; Patton et al. 2014; Chen et al. 2016). These outstanding activities together with the intriguing chemical structures of flavaglines, characterized by a densely functionalized tricyclic nucleus with five contiguous stereocenters, attracted considerable synthetic interest. Starting with the first total synthesis of rocaglamide (**25**) (Trost et al. 1990), Zhao et al. (2016) provided an overview about the various synthetic approaches to the total synthesis of flavaglines.

Apart from the successful development of synthetic analogues with high antiproliferative properties (Gerard et al. 2004; Thede et al. 2004; Adams et al. 2009; Thuaud et al. 2009, 2011; Roche et al. 2010; Rodrigo et al. 2012; Liu et al. 2012; Lajkiewicz et al. 2012; Hawkins et al. 2014; Chu et al. 2019; Maïga et al. 2019; Chan et al. 2019; Harmouch et al. 2020; Yan et al. 2020; Ernst et al. 2020), further studies on structural diversification and distribution of naturally occurring flavaglines are greatly hampered by taxonomic problems especially in the complex species in *Aglaia* (Pannell 1992). As a consequence, published reports on flavaglines are frequently attributed to the wrong species and have resulted in confusion in the chemical and pharmaceutical literature. This makes it difficult to compare the results of the different research groups, to replicate their work or to use the data for chemotaxonomic conclusions*.* Relying on expert identification by Caroline Pannell from the University of Oxford broad-based phytochemical comparisons in our laboratory exhibited characteristic biogenetic trends which already contributed as chemical markers together with morphological characters and DNA sequence data to a more natural taxonomic grouping (Brem 2002; Muellner et al. 2005, 2009). The present review provides a visual reference guide to the structural diversity of naturally occurring flavaglines, including all newly described derivatives. On the basis of biosynthetic considerations and the coexistence of characteristic substitution patterns 148 flavaglines were grouped into 77 benzofurans, 61 benzopyrans, and 10 benzoxepins. Different accumulation trends and structural variation were compared with regard to the presently suggested species delimitation and grouping in *Aglaia*. In addition, the purpose of this article is to give an updated summary of the numerous publications demonstrating the wide range of bioactivities of this fascinating class of compounds.

## Structural relationships

**Fig. 1:**
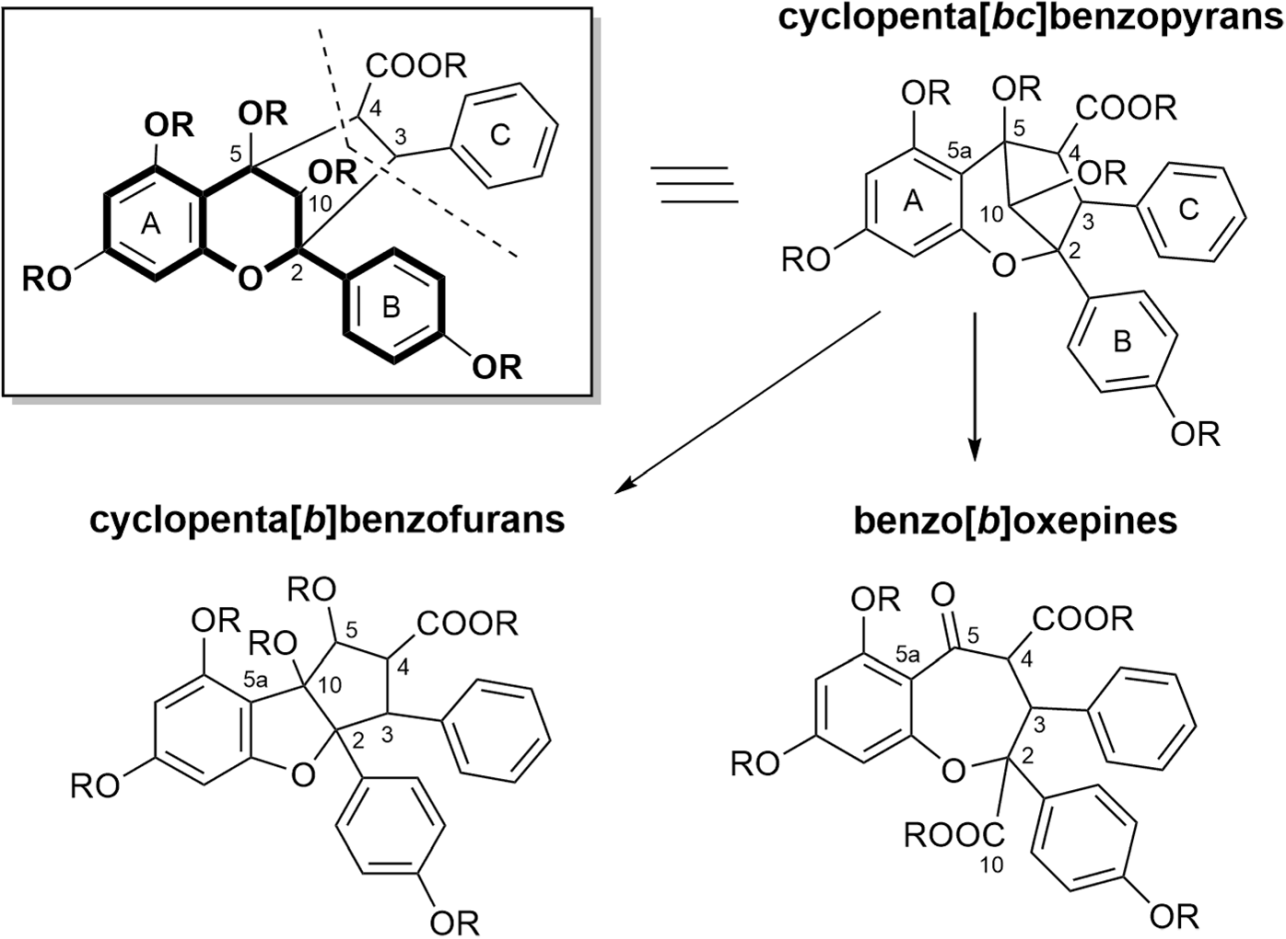
Proposed biogenetic correlation between flavagline skeletons (note the numbering of the benzopyran ring is used here for all structures) (Bacher et al. 1999)

Although the biosynthesis of flavaglines is not yet confirmed experimentally, the biogenetic correlations of three basic skeletons shown in Fig. 1 are widely accepted. According to that a flavonoid nucleus is linked to a cinnamic acid moiety resulting in the formation of a cyclopenta[*bc*]benzopyran skeleton which represents a biosynthetic key intermediate to both the cyclopenta[*b*]benzofurans and benzo[*b*]oxepines (Bacher et al. 1999; Nugroho et al. 1999; Kim et al. 2006b). This hypothesis is supported by the occurrence of common substitution patterns of the aromatic rings A and B in all three skeletal types and in the co-occurring flavonoids (Brem 2002). The formation of the benzofuran skeleton can be explained by opening the 5–5a bond of the benzopyran precursor and closing the 5a–10 bond, whereas the benzoxepines may be derived from the benzopyrans by a cleavage of the 10–5 bond (Fig. 1). The stereochemical implications created by these conversions were discussed by Bacher et al. (1999) and Proksch et al. (2001).

### Bisamides

**Fig. 2:**
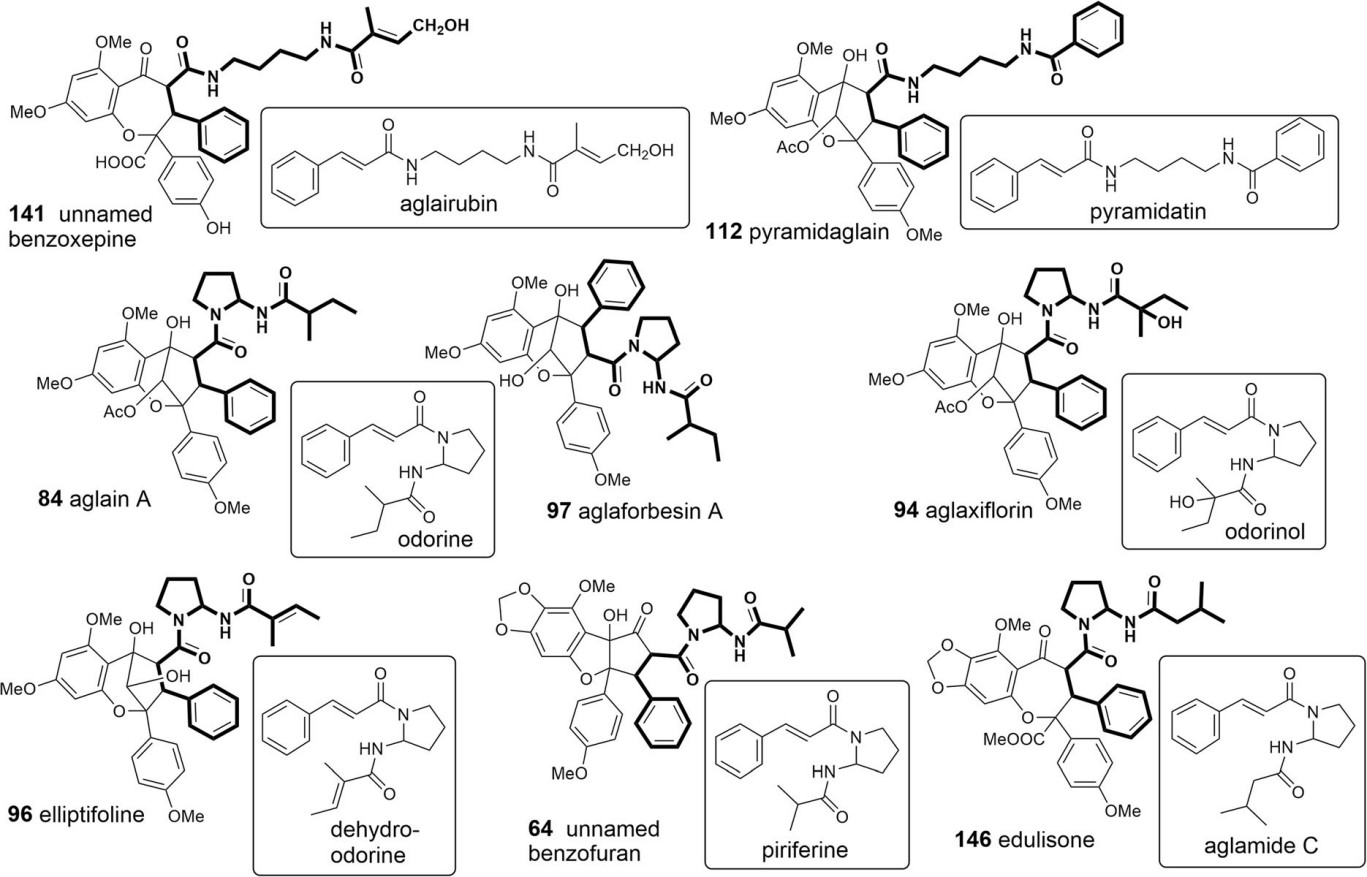
Various bisamides as building blocks of flavaglines (Greger et al. 2008)

The occurrence of flavaglines was shown to be closely correlated with the formation of putrescine-derived bisamides representing both characteristic constituents of the leaf extracts of *Aglaia* species as well as important building blocks of many flavaglines (Fig. 2). Structurally, bisamides were found to occur either as cyclic 2-aminopyrrolidine derivatives (Table 1A) or in an open-chained form (Table 1B). The former were shown to have a more restricted distribution so far only known from the genus *Aglaia* (Detterbeck and Hesse 2002). Besides a few derivatives containing two identical acid moieties the majority of bisamides is characterized by two different acid residues with cinnamic acid as the most frequent acid part. Following the supposed biogenetic correlations outlined in Fig. 1, it represents the structural prerequisit for the incorporation of bisamides into flavaglines. As shown in Table 1A and B the cinnamic acid-derived bisamides are frequently linked with 2-methyl butyric acid as second acid part which shows further variation by different hydroxylations, dehydrations (tiglic acid), and stereochemistry (Hayashi et al. 1982; Duh et al. 1993; Saifah et al. 1993; Duong et al. 2007). In addition, isobutyric, isovaleric, senecioic, phenylacetic, and benzoic acid moieties were also reported. In *A. leucophylla* King, published as *A. leptantha* Miq. (Greger et al. 2000), and *A. edulis* (Roxb.) Wall. (Saifah et al. 1999; Kim et al. 2006a) bisamides were shown to be also linked to the rare methylthiopropenoic acid. However, to date no sulfur-containing bisamides have been reported as part of flavaglines. The threefold acylated putrescine derivative edulimide was isolated as a major component from the leaf extract of *A. edulis* collected in SE-Thailand. In addition to cinnamic and dihydrocinnamic acid edulimide contains an acetic acid moiety forming an imide group (Brader et al. 1998) (Table 1B). The discovery of structurally corresponding amide esters in *A. tenuifolia* Hiern let expect close biogenetic connections to bisamides. In this case one nitrogen atom has obviously been replaced with oxygen (Greger et al. 2008).

**Table 1:**
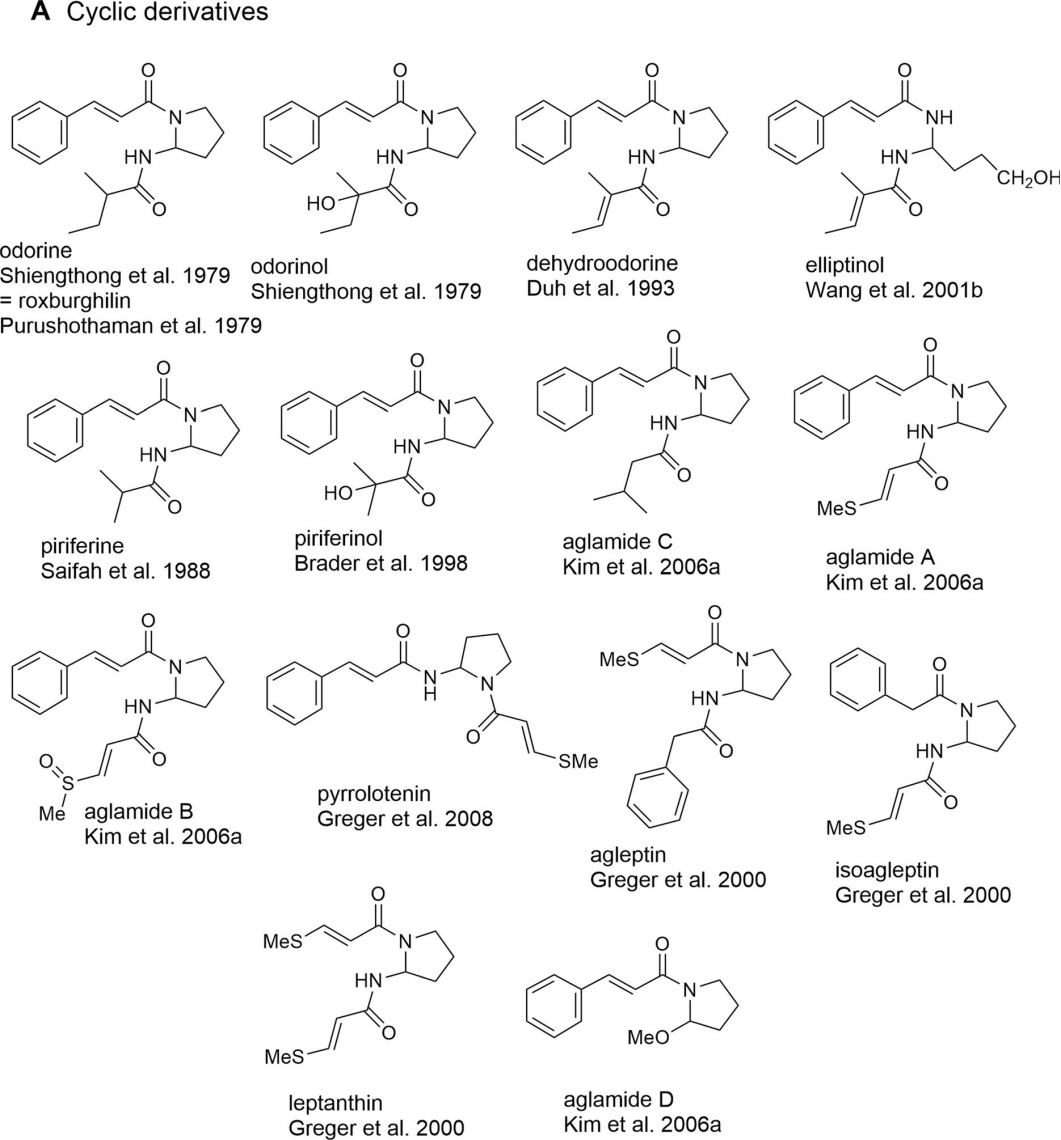

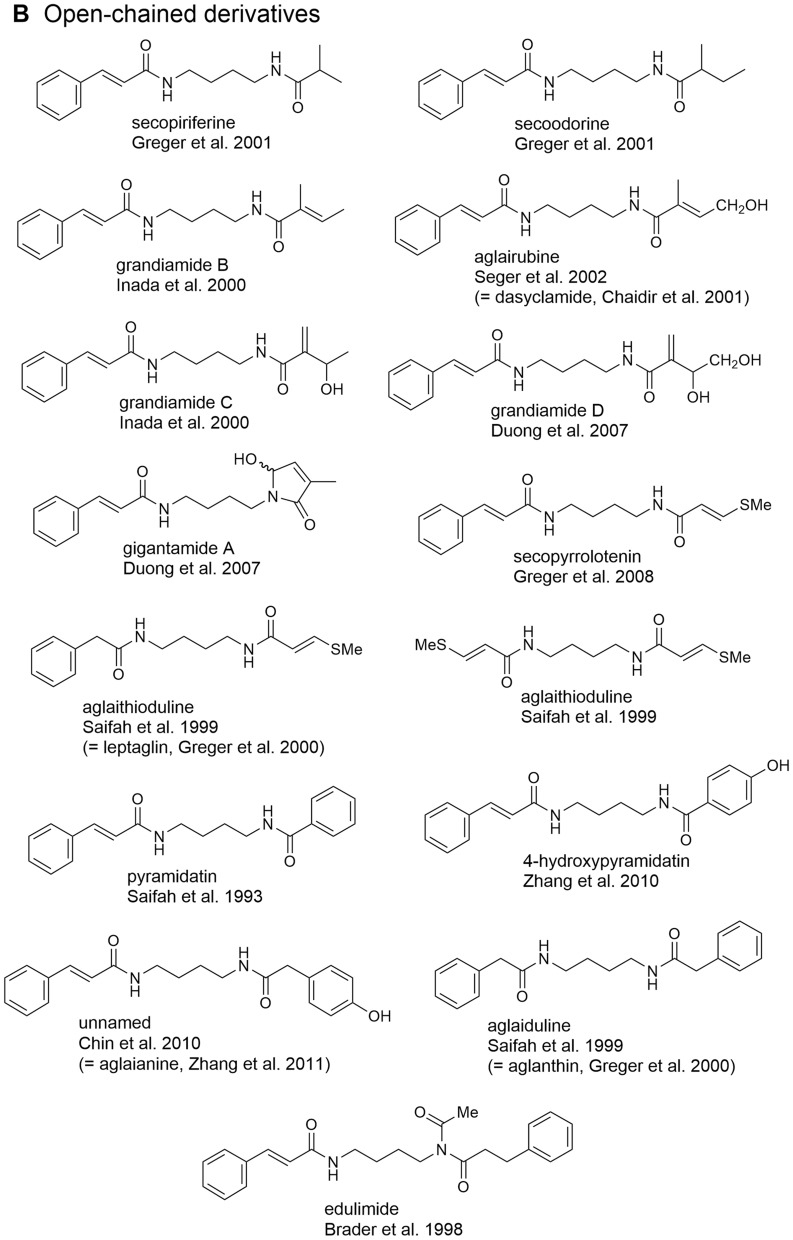
Putrescine-derived bisamides from *Aglaia* species

Based on the flavagline structures reported so far it became evident that putrescine-derived bisamides are essential building blocks mainly incorporated into benzopyran and benzoxepine derivatives. In this case both open-chained and cyclic bisamides are inserted as a whole maitaining their overall structure and substitution pattern. This became especially clear e.g. with the formation of aglain A (**84**) and aglaforbesin A (**97**), where odorine is incorporated in two different positions (Fig. 2).

### Benzofuran flavaglines

**Fig. 3:**
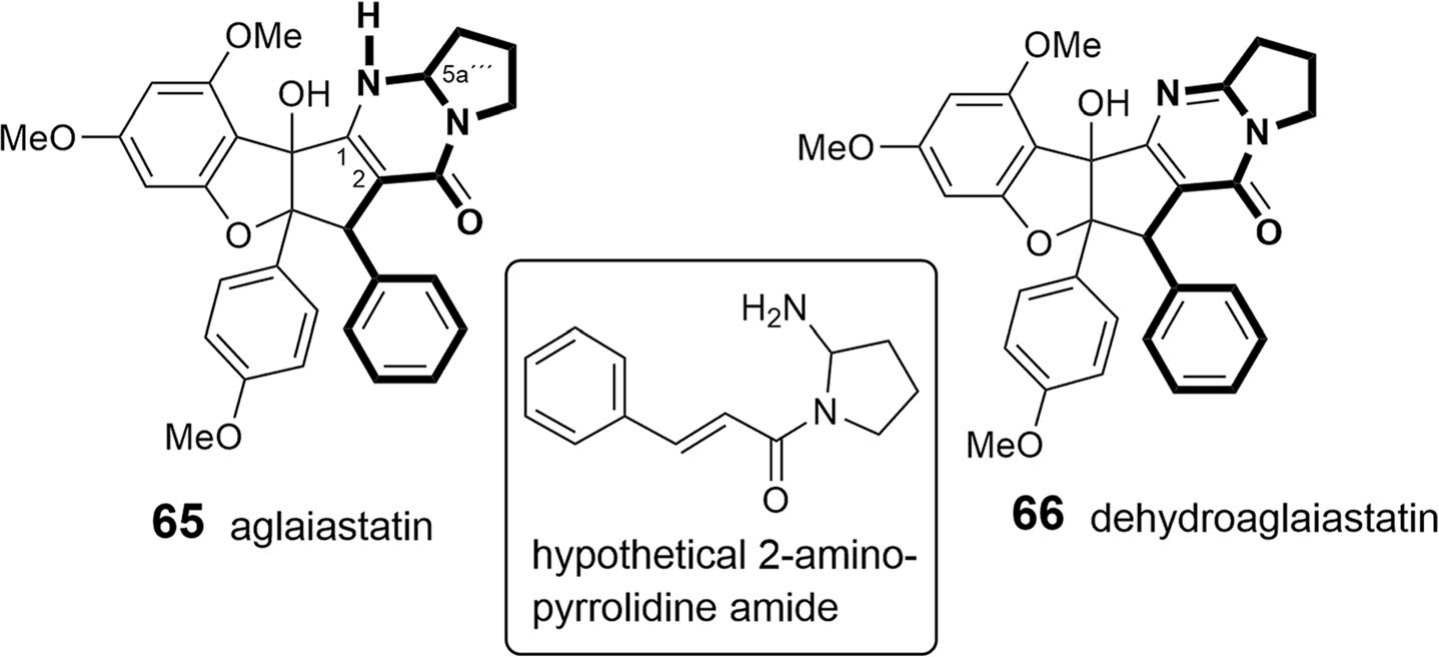
Formation of pyrimidinones with a hypothetical putrescine-derived 2-aminopyrrolidine amide (Greger et al. 2008)

**Fig. 4:**
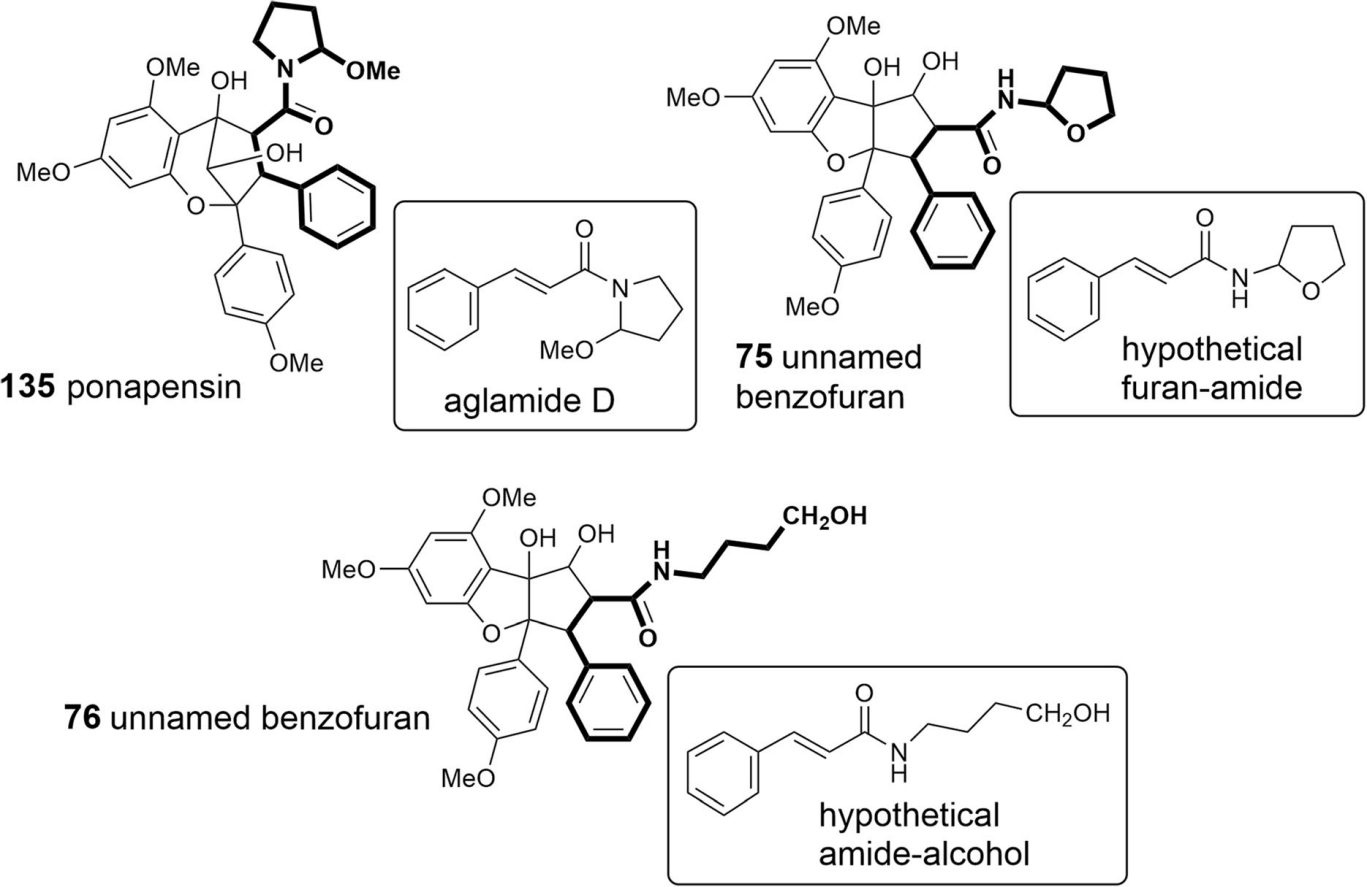
Benzopyran- and benzofuran-type flavaglines with amide-alcohol derived moieties

As shown in Tables 2, 3, and 4, putrescine-derived bisamides are mostly linked to benzopyran and benzoxepine flavaglines, but were also detected in the unnamed benzofuran **64** (Dreyer et al. 2001)**.** A hypothetical putrescine-derived aminopyrrolidine amide was suggested to form a series of pyrimidinones (**65**–**74**). In this case the pyrimidinone moiety was speculated to be created by a condensation reaction between the *NH*_2_-group of the amide with the *OH*-group at *C*-1 of the benzofuran moiety (Fig. 3) (Greger et al. 2008). From ten pyrimidinones listed in Table 2 the three derivatives aglaiastatin (**65**) (Ohse et al. 1996), aglaroxin I (**71**), and aglaroxin H (**73**) (Molleyres et al. 1999) deviate by a reduced bond between *C*-5a′′′ and *N*. It is interesting to note that aglaiastatin (**65**), isolated from *A. gracilis* A.C. Smith, was converted into dehydroaglaiastatin (**66**) during purification by preparative TLC (Greger et al. 2001; Hausott et al. 2004). This indicates lower stability of compound **65** and possibly explains the more frequent reports on **66** and other corresponding dehydro-derivatives. Although no amide-esters have been found so far as building blocks of flavaglines, closely related derivatives have been reported where amide-alcohol derived moieties are incorporated into benzofurans (**75**–**77**). In this case one nitrogen atom of putrescine has obviously been replaced with oxygen as assumed for the aforementioned amide-esters isolated from *A. tenuifolia* (Fig. 4) (Greger et al. 2008).

**Table 2:**
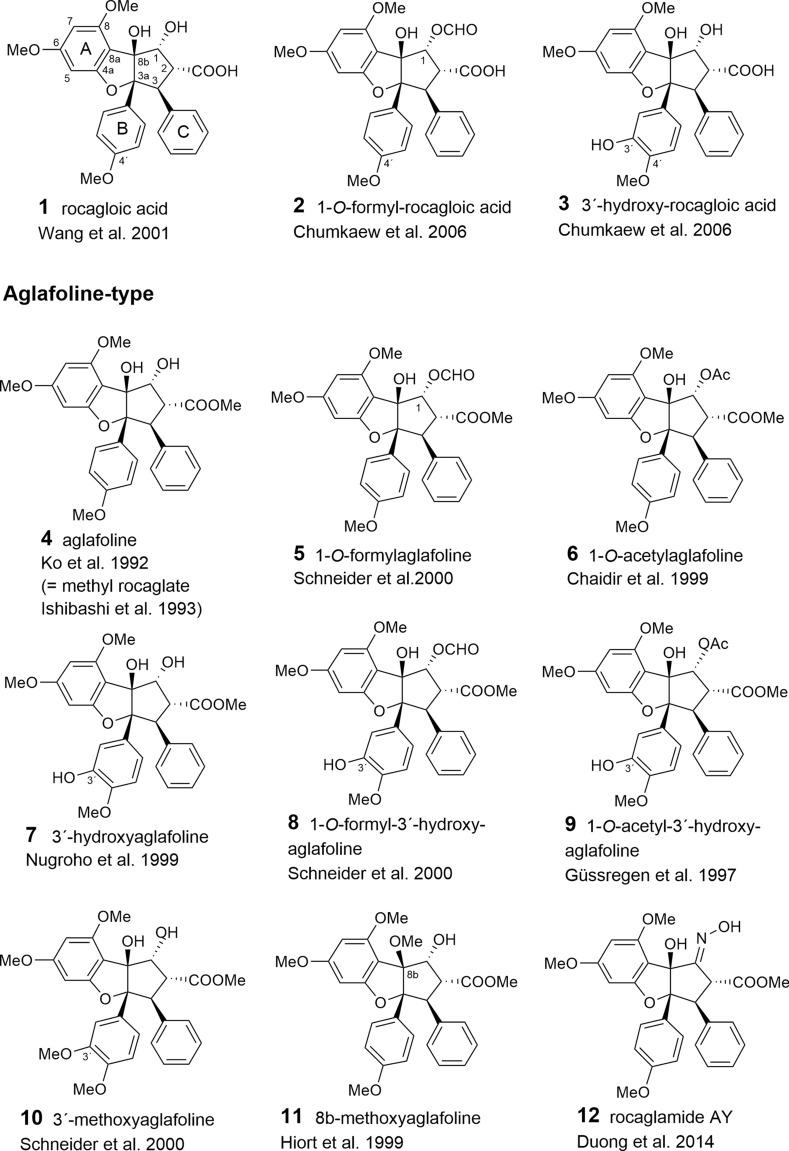

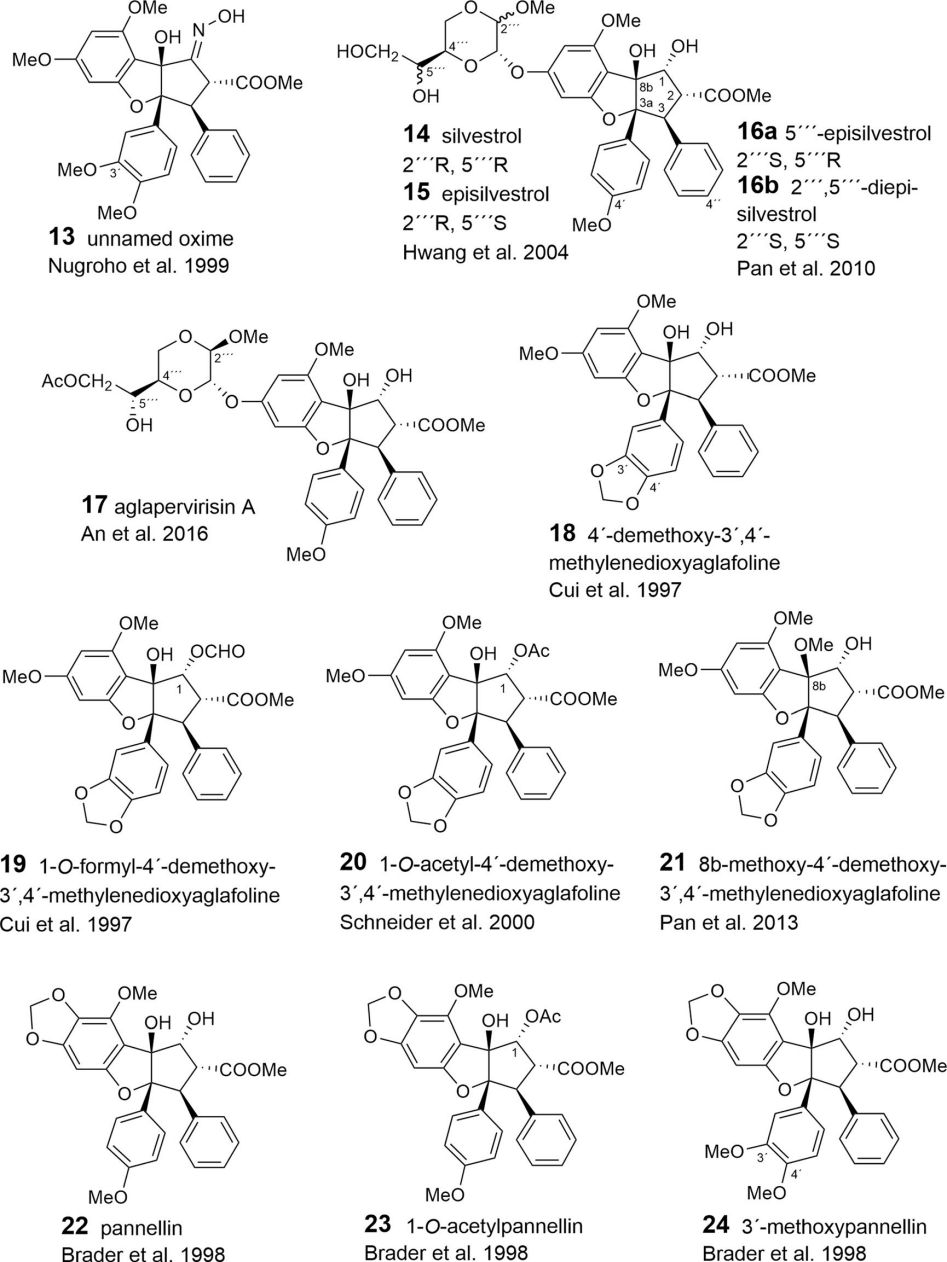

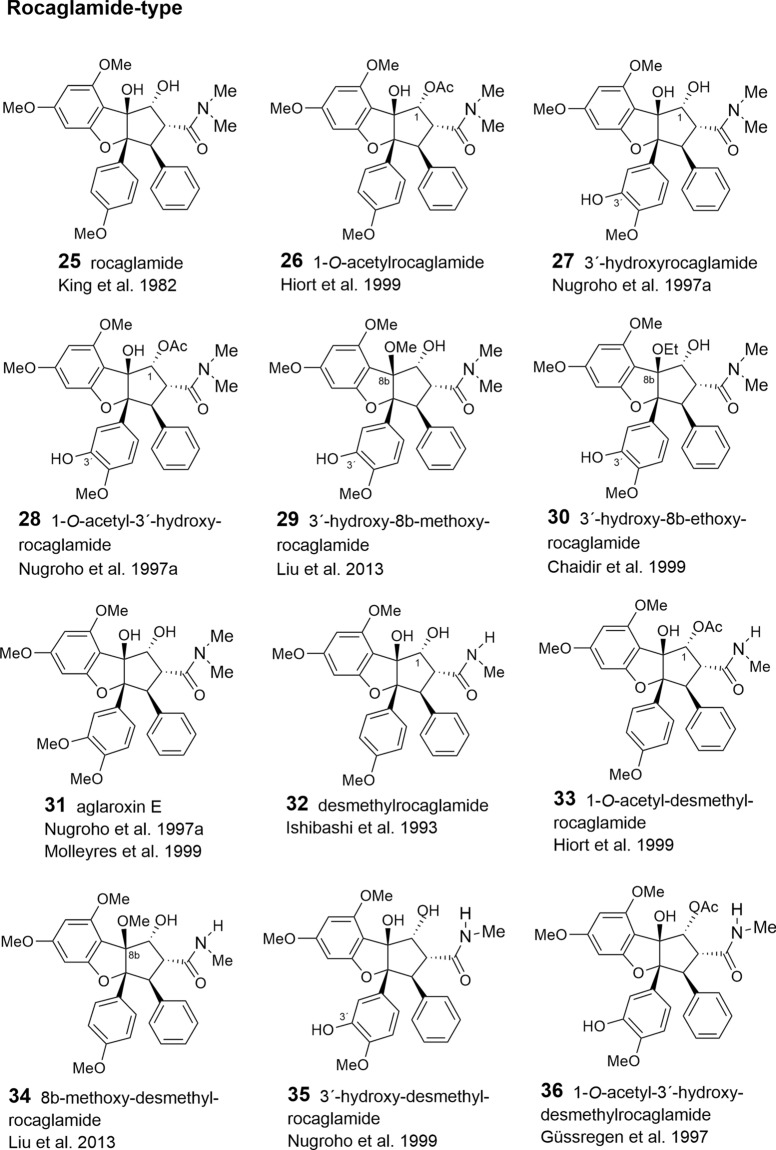

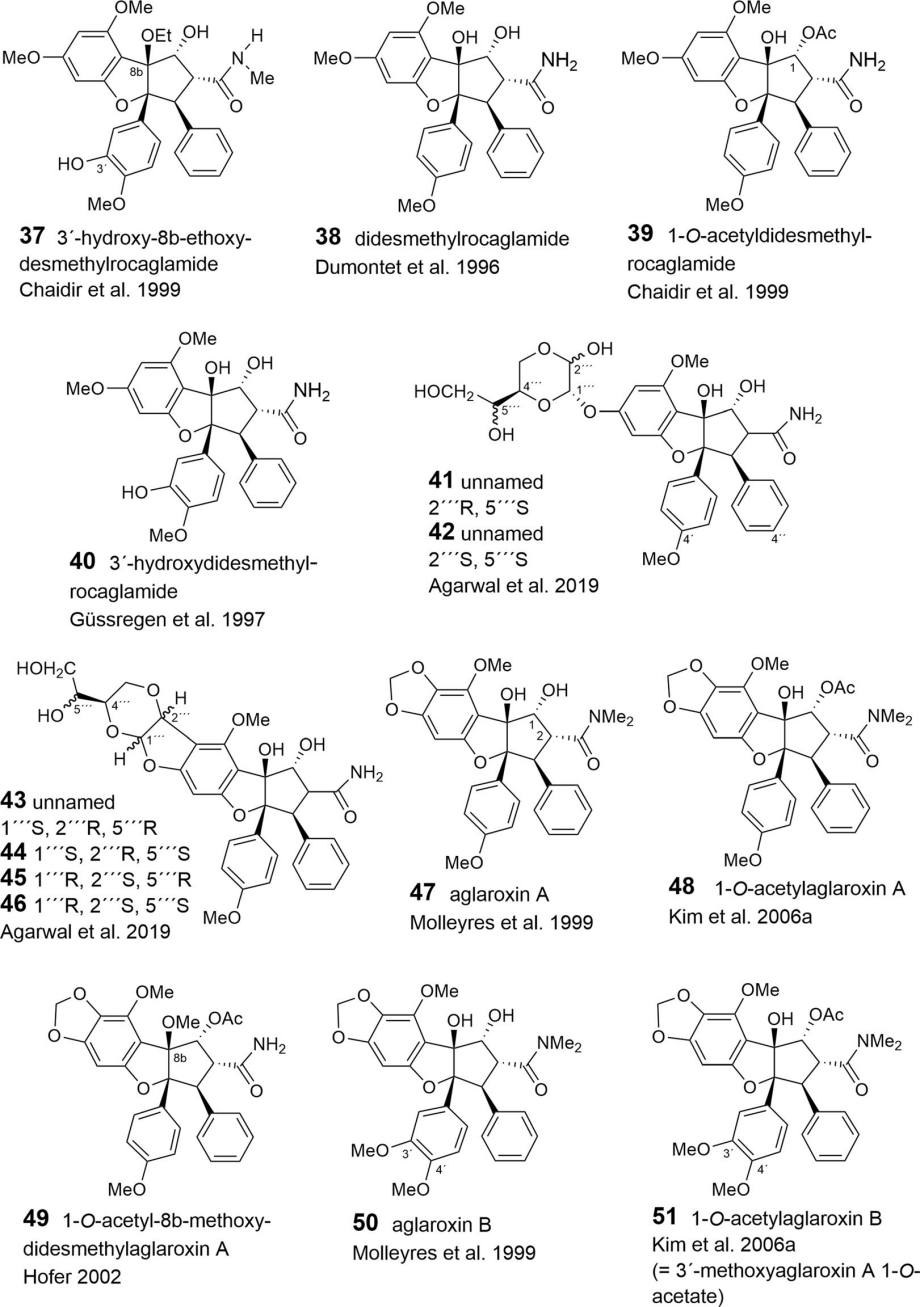

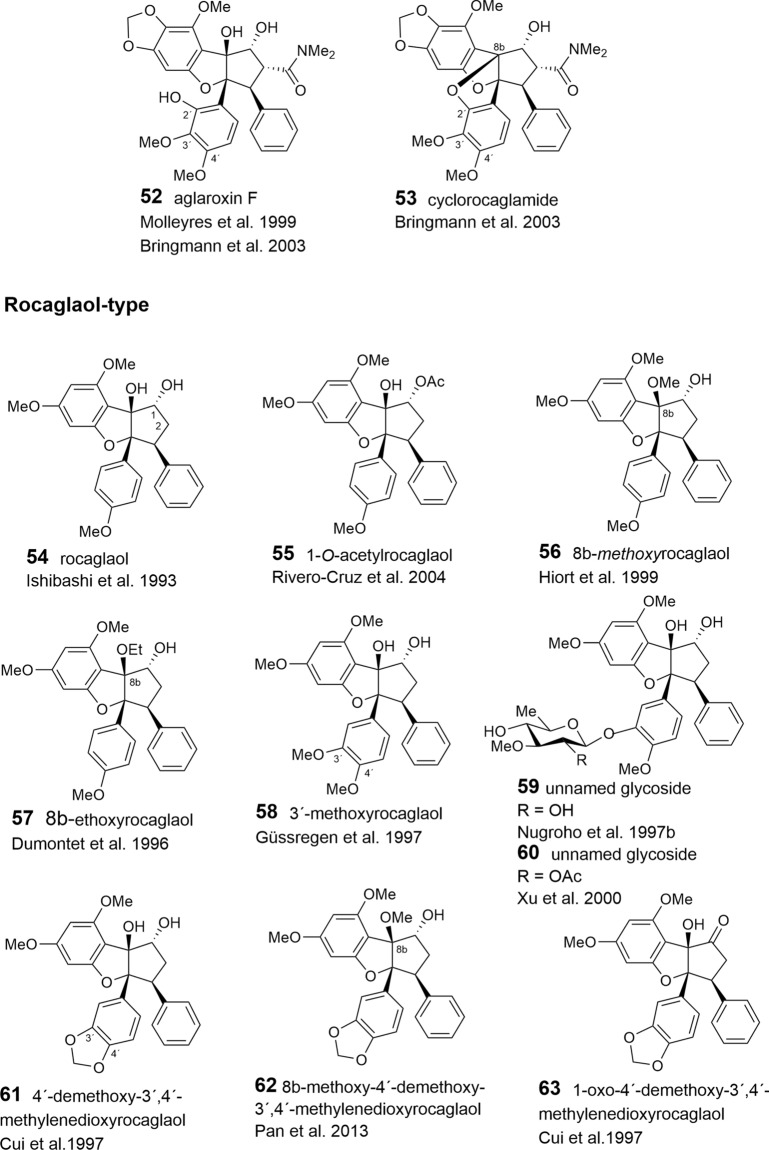

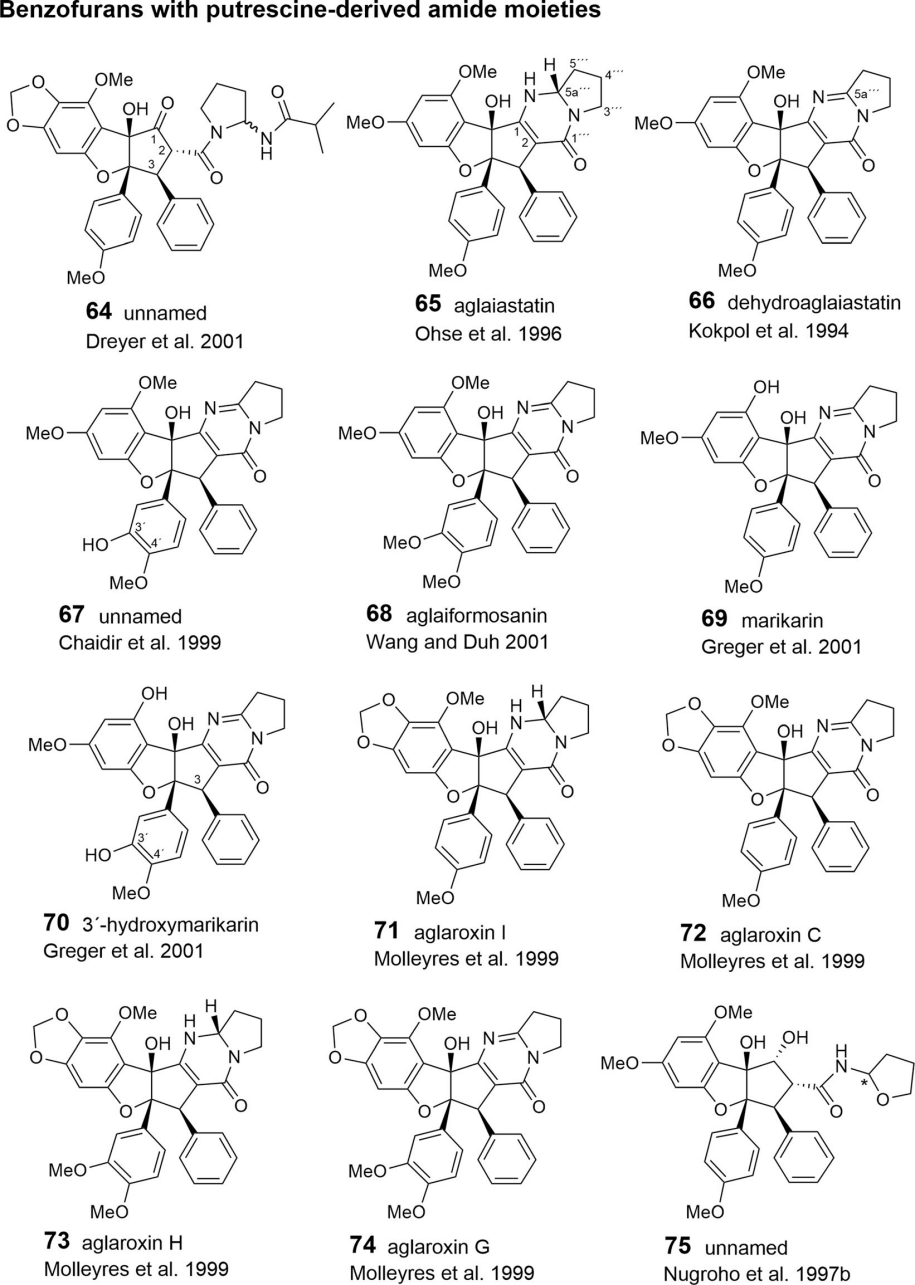

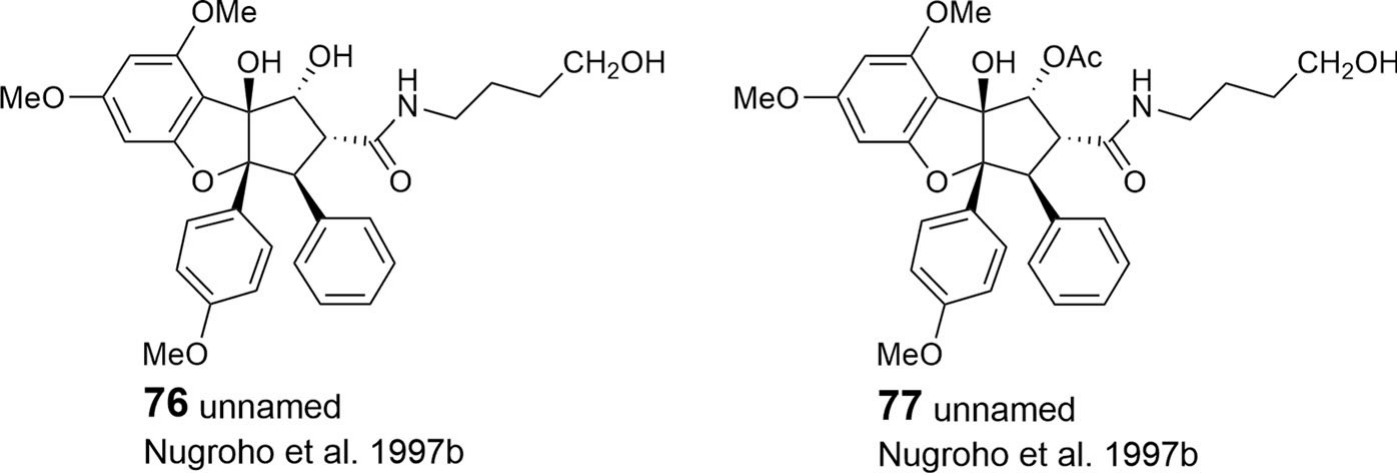
Benzofuran flavaglines

**Table 3:**
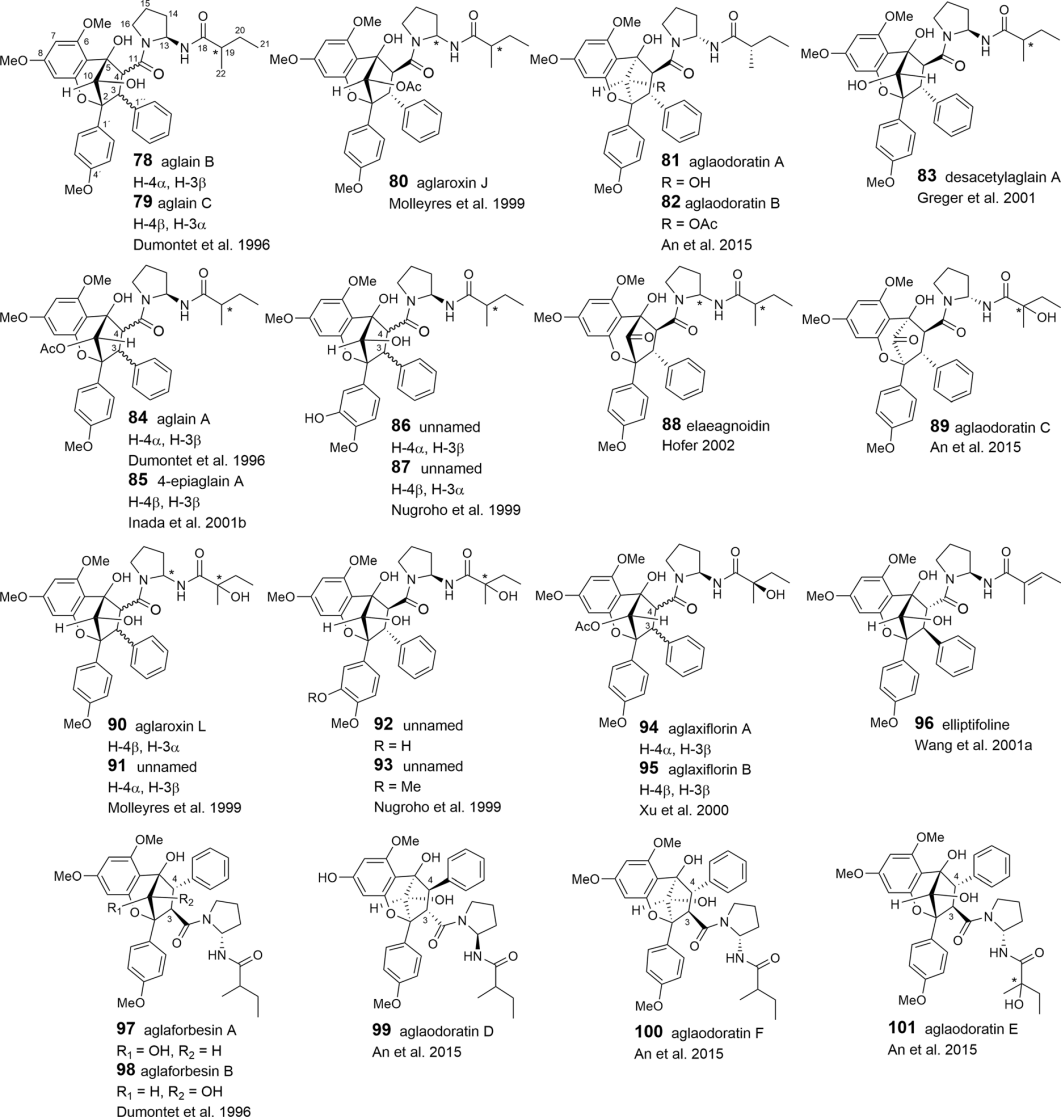

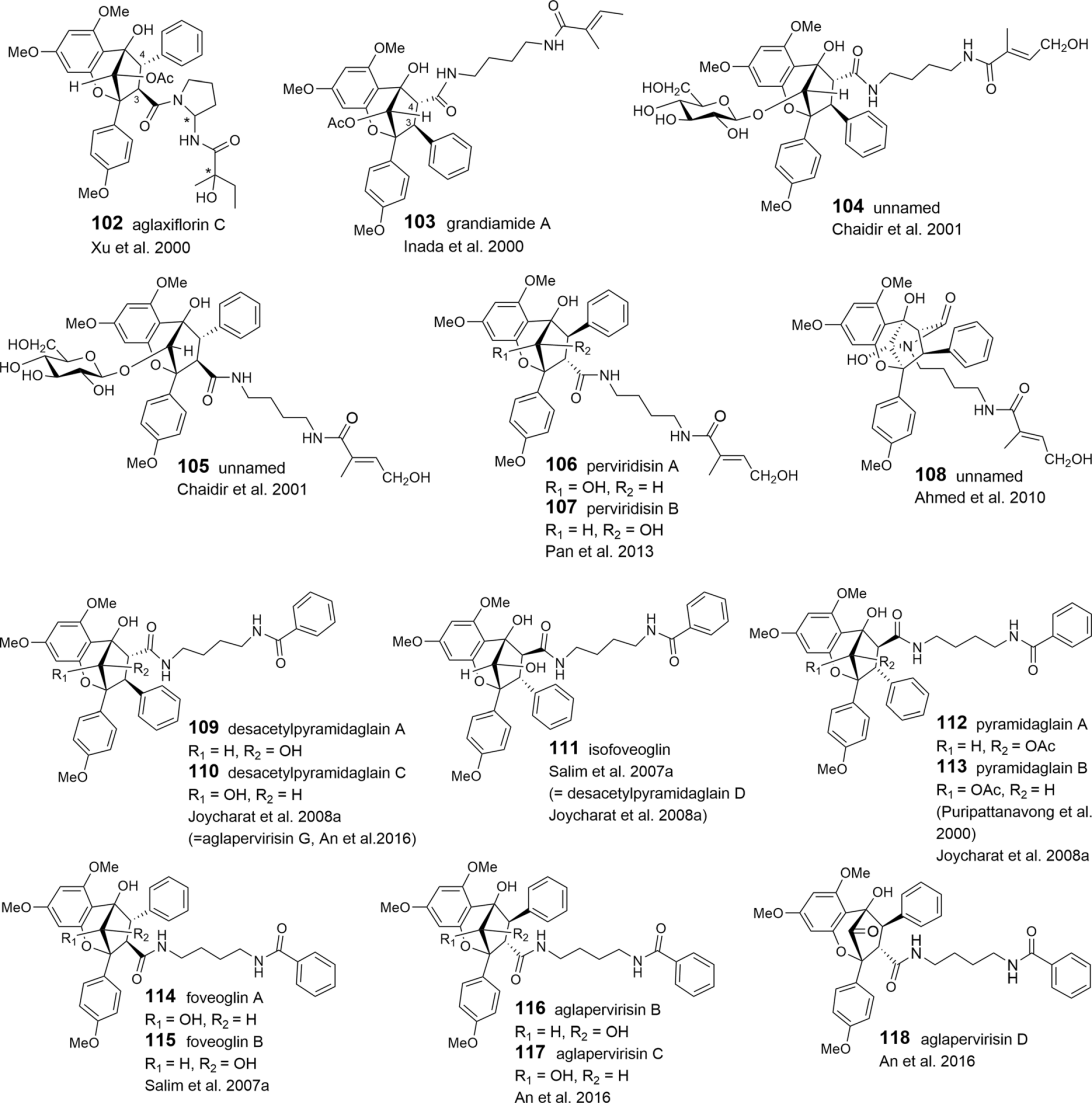

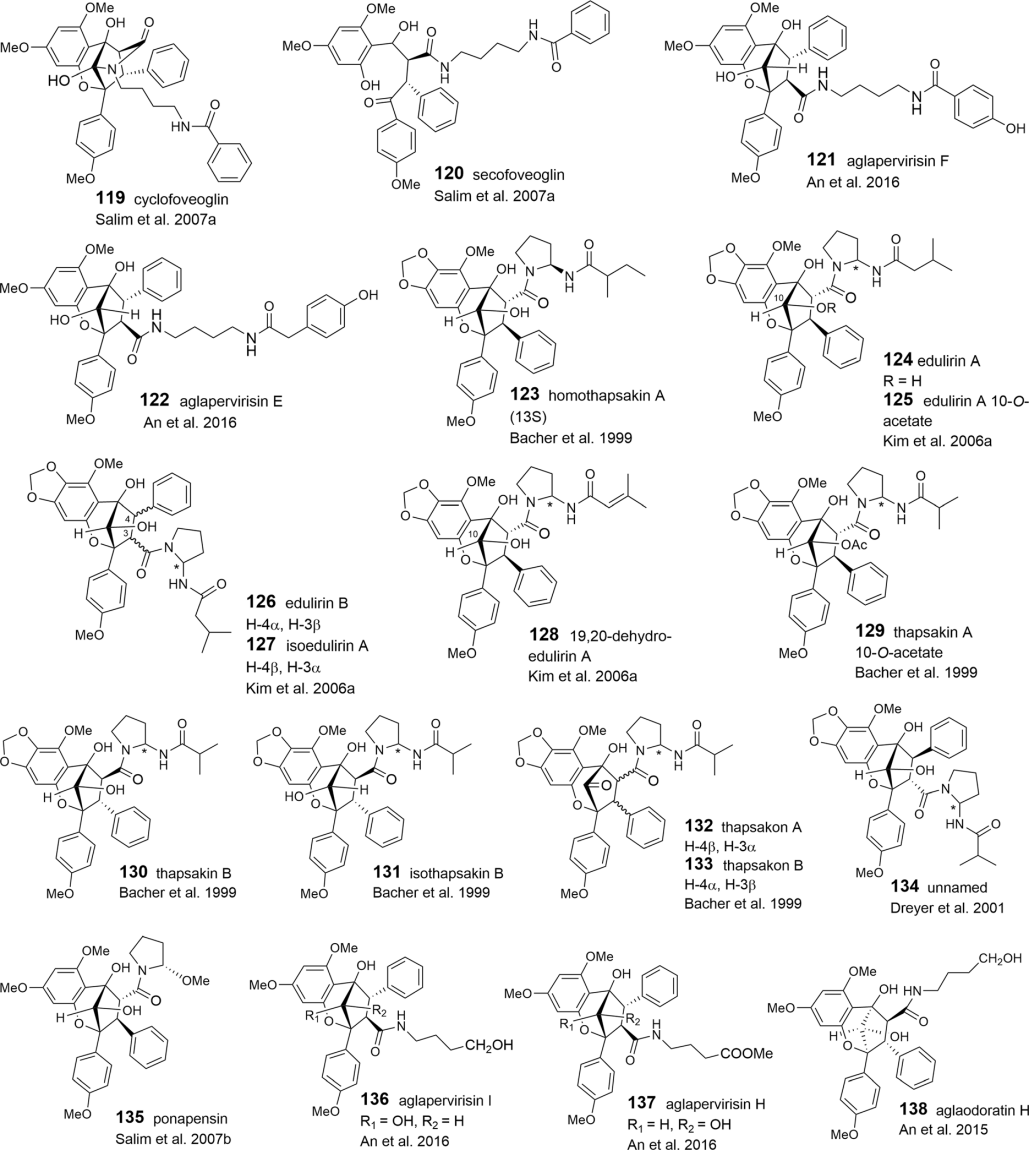
Benzopyran flavaglines

*Epimers not determined

**Table 4:**
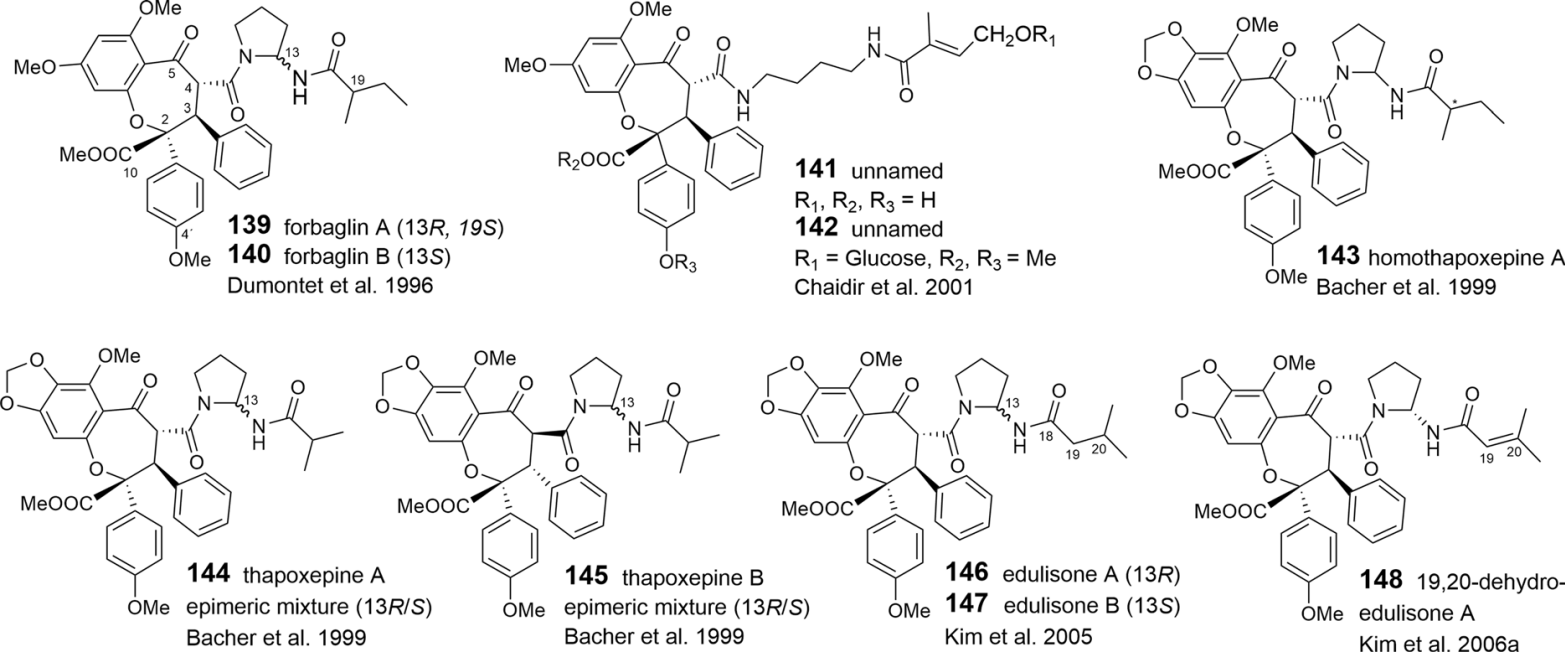
Benzoxepines

With respect to the presumed central role of cinnamic acid in the formation of the flavagline basic skeleton (Fig. 1) rocagloic acid (**1**) and its congeners **2** and **3** may be regarded as biosynthetic precursors from which both the aglafoline-type^2^ (**4**–**24**) and the nitrogen-containing rocaglamide-type benzofurans (**25**–**53**) can be derived. Consequently, the rocaglaol-type derivatives (**54**–**63**) can be considered as the result of decarboxylation at *C*-2 (Table 2). The before mentioned fourth type of benzofurans is characterized by an incorporation of putrescine-derived amide moieties leading to the unnamed compounds **64, 75**–**77** and the pyrimidinones **65**–**74**. Different methylations of the amino-group in the rocaglamide-type flavaglines result in a series of corresponding desmethyl (**32**–**37**) and didesmethyl derivatives (**38**–**40**, **49**). Further grouping within the benzofurans is suggested by different substitution patterns of aromatic ring A, showing either the two widespread methoxy groups in *meta*-position at *C*-6 and *C*-8, or a methylenedioxy group at *C*-6, *C*-7 combined with a methoxy group at *C*-8. The latter substitution pattern appears to be typical for a group of probably closely related *Aglaia* species and was also found in benzopyran and benzoxepine flavaglines (Tables 3, 4). Of special interest was the discovery of the unique structure of silvestrol (**14**) in the fruits and twigs of *A. foveolata* Pannell, published as *A. silvestris* (M. Roemer) Merrill. It deviates from the other flavaglines by an unusual dioxanyloxy moiety replacing the methoxy group at *C*-6 (Hwang et al. 2004). An acetylation of the *C*-6′′′ OH group of the dioxanyl ring was reported for aglapervirisin A (**17**) isolated from the leaf exract of *Aglaia perviridis* Hiern (An et al. 2016). Modified analogues of silvestrol (**14**) with a *CONH*_2_ group at *C*-2 (**41**, **42**) were recently isolated from this species along with four related derivatives, which are characterized by a new carbon skeleton with a fused 2,3-dihydrobenzofuran unit attached to the dioxanyl ring (**43**–**46**) (Agarwal et al. 2019). Another unusual substitution of ring A is the replacement of the *C*-8 methoxy group by a hydroxy group in the marikarins (**69**, **70**), only known so far from *A. gracilis* (Greger et al. 2001). The substitution pattern of aromatic ring B is mostly characterized by one methoxy group in *para*-position at *C*-4′, frequently accompanied by hydroxylation or methoxylation at *C*-3′, and sometimes, by the formation of a methylenedioxy group at *C*-3′, *C*-4′. A threefold substitution of ring B at C-2′, C-3′, C-4′ was reported only for aglaroxin F (**52**), isolated from *A. elaeagnoidea* (A. Juss.) Benth. (= *A*. *roxburghiana* (Wight & Arn.) Miq.) (Molleyres et al. 1999), and for cyclorocaglamide (**53**) from *A. oligophylla* Miq. (Bringmann et al. 2003). The latter represents the first flavagline with an oxygen bridge between *C*-8b and *C*-2′ of the aromatic ring B.

An important structural variation in the benzofurans is created by different substitutions of the hydroxy groups at *C*-1 and *C*-8b of the cyclopentane ring. While the former is frequently esterified with acetyl- (**6**, **9**, **20**, **23**, **26**, **28**, **33**, **36**, **39**, **48**, **49**, **51**, **55**, **77**) or formyl-residues (**2**, **5**, **8**, **19**), the latter can be substituted by a methoxy- (**11**, **21**, **29**, **34**, **49**, **56**, **62**) or ethoxy-group (**30**, **37**, **57**). Rare chemical features are represented at *C*-1 by the formation of oximes in the unnamed derivative **13** (Nugroho et al. 1999) and in rocaglamide AY (**12**) (Duong et al. 2014), or by an insertion of an oxo-group in **63** (Cui et al. 1997) and **64** (Dreyer et al. 2001). Glycosylation has been reported so far only for the hydroxy group at C-3′ of aromatic ring B in the unnamed derivatives **59** (Nugroho et al. 1997b) and **60** (Xu et al. 2000).

### Benzopyran and benzoxepine flavaglines

The structural variation of the benzopyrans and benzoxepines is mainly formed by different bisamide parts attached to the basic skeleton. Apart from cyclized or open-chained putrescine moieties (Table 1A, B), the differences are created by different acid residues. As shown in Tables 3 and 4, derivatives with 2-methyl butyric acid are dominating in the benzopyrans **78**–**108** and benzoxepines **139–143**, displaying additional variation by hydroxylation and dehydration (tiglic acid) of the acid part. Isobutyric (**129**–**134**, **144**, **145**), isovaleric (**124**–**127**, **146**, **147**), and senecioic acid residues (**128**, **148**) were also found in benzopyrans and benzoxepines, where they are linked to cyclized putrescine only. By contrast, benzoic acid residues are uniformly linked to open-chained putrescine only found in the benzopyrans **109**–**120**. Aglapervirisins F (**121**) and E (**122**), isolated from the leaves of *A. perviridis* Hiern, differ by *para*-hydroxylated benzoic or phenylacetic acid parts, respectively (An et al. 2016). In the coexisting aglapervirisins I (**136**) and H (**137**), as well as in ponapensin (**135**) (Salim et al. 2007b), the putrescine derived bisamides are replaced by other residues presumably derived from amide-alcohol precursors (Fig. 3).

Apart from the two different substitution patterns of aromatic ring A, already mentioned for the benzofurans, additional variation in the benzopyrans and benzoxepines is created by different configurations at *C*-3 and *C*-4, and substitutions and stereochemistries of the hydroxy group at *C*-10 in the benzopyrans. Rare structural transformations are featured by cyclofoveoglin (**119**), isolated from the stem bark of *A. foveolata* (Salim et al. 2007a), and the unnamed benzopyran **108** from the leaves of *A. cucullata* (Roxb.) Pellegrin (Ahmed et al. 2010). Both are formed by a cyclization reaction between *N*-12 of the bisamide moiety and *C*-10 of the basic skeleton. Ring-opening leads to the formation of secofoveoglin (**120**), which may also be interpreted as a benzoxepine derivative (Salim et al. 2007a). Glycosylation has been reported for the two benzopyrans **104** and **105**, and the benzoxepine **142**, which were isolated from the leaf extract of the Chinese *A. dasyclada* Miq. From the same extract the related derivative **141** was shown to differ from **142** by the absence of the glucose moiety and two methoxyl groups at *C*-4′ and *C*-10 (Chaidir et al. 2001)**.**

## Bioactivities

### Insecticidal activities

**Fig. 5:**
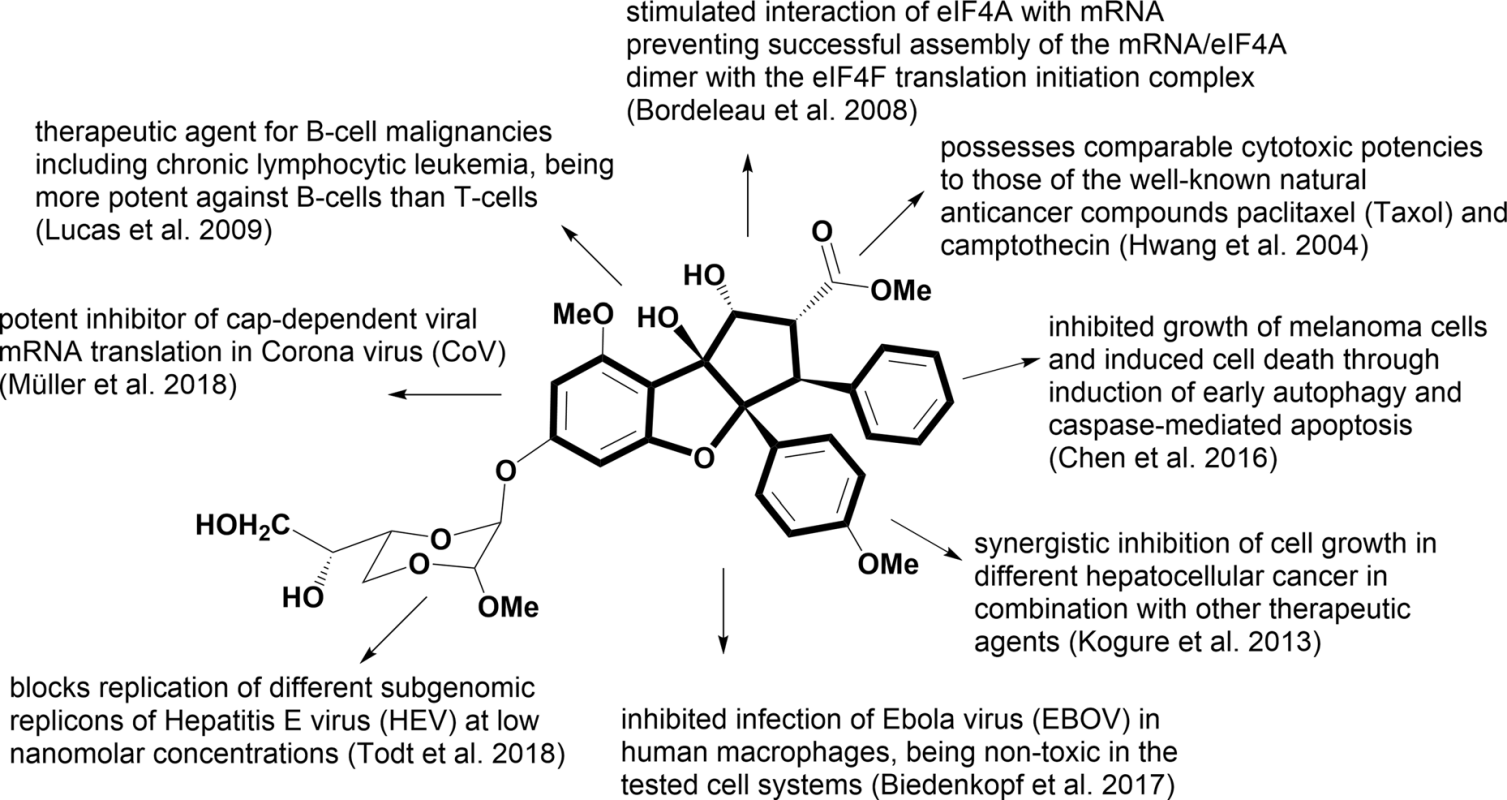
Anticancer and antiviral activities of silvestrol (**14**)

The prerequisite for the high insecticidal activity of flavaglines was the formation of a cyclopentabenzofuran basic skeleton. In contrast, benzopyran and benzoxepine flavaglines did not show significant effects (Bacher et al. 1999; Nugroho et al. 1999; Molleyres et al. 1999; Dreyer et al. 2001). The first four active derivatives, rocaglamide (**25**), desmethylrocaglamide (**32**), aglafoline (= methyl rocaglate) (**4**), and rocaglaol (**54**), were obtained by bioassay-guided isolation from the twigs and leaves of *A. odorata* Lour. (Janprasert et al. 1993; Ishibashi et al. 1993). In order to investigate the insecticidal and anti-feeding properties of various derivatives and to determine structure–activity relationships a representative number of benzofurans were tested in the following studies against the polyphagous pest insect *Spodoptera littoralis*. The very high activity of didesmethylrocaglamide (**38**) with survival rate (LC_50_) and growth inhibition (EC_50_) values at 0.8 ppm and 0.05 ppm, respectively, was comparable with that of the well-known natural insecticide azadirachtin with 0.9 ppm and 0.04 ppm (Nugroho et al. 1997b). On the basis of comparative feeding experiments it became apparent that especially different substitutions of the *OH*-groups at *C*-8b and *C*-1 of the cyclopentane ring are essential for insecticidal activity. As shown in Fig. 5 acetylation at *C*-1 reduced the LC_50_ value of rocaglamide (**25**) from 0.9 ppm to 7.1 ppm, and of aglafoline (**4**) from 1.3 ppm to 6.62 ppm. This reducing effect by substituting the *OH*-group at *C*-1 by an *O*Ac-group was also shown in the related derivatives **9**, **33**, and **36**, which were approximately sevenfold less active than their corresponding non-acetylated derivatives (Nugroho et al. 1997a; Güssregen et al. 1997; Hiort et al. 1999; Chaidir et al. 1999), and was also confirmed for 1-*O*-acetylpannelline (**23**) with a different substitution pattern in aromatic ring A (Brader et al. 1998). Similarily, the two *C*-1 formylated derivatives **5** and **8** were shown to be five times less active than the corresponding non-substituted derivatives **4** and **7** (Schneider et al. 2000). An even stronger effect was observed by replacing the *OH*-group at *C*-8b. After substitution by a methoxy-group in 8b-methoxyaglafoline (**11**) and 8b-methoxyrocaglaol (**56**) no activity could be determined in both compounds up to a cocentration of 100 ppm. This intriguing effect was also confirmed for the derivatives **30** and **37** containing an ethoxy-group at *C*-8b (Chaidir et al. 1999). While the different substituents at *C*-2, leading to ester (aglafoline-type) or amide groups (rocaglamide-type), revealed only marginal influences, the unsubstituted *C*-2 (rocaglaol-type) showed a clear decrease of activity (Fig. 5) (Hiort et al. 1999; Bacher et al. 1999; Schneider et al. 2000; Ebada et al. 2011).

Modifications of the substitution pattern of aromatic ring A showed higher activity for compounds with dimethoxylation at *C*-6 and *C*-8 than those with a *C*-6, *C*-7 methylenedioxy substitution combined with a methoxy-group at *C*-8 (Bacher et al. 1999; Molleyres et al. 1999; Dreyer et al. 2001). These two substitution patterns were shown to lead to a different mode of action in the otherwise identical derivatives rocaglamide (**25**) and aglaroxin A (**47**) (Koul et al. 2004, 2005). The replacement of the *C*-8 methoxy-group of aglaiastatin (**65**) by a hydroxy-group in marikarin (**69**) reduced the LC_50_ value from 1.2 to 12.2 ppm (Greger et al. 2001). A reduction of activity was also observed by additional substitutions in aromatic ring B (Molleyres et al. 1999; Schneider et al. 2000). Preparation of synthetic benzofurans showed that analogues of rocaglamide (**25**) with equal or improved insecticidal activity could not be achieved (Li et al. 2008; Hall et al. 2017) (Table 5).

**Table 5:**
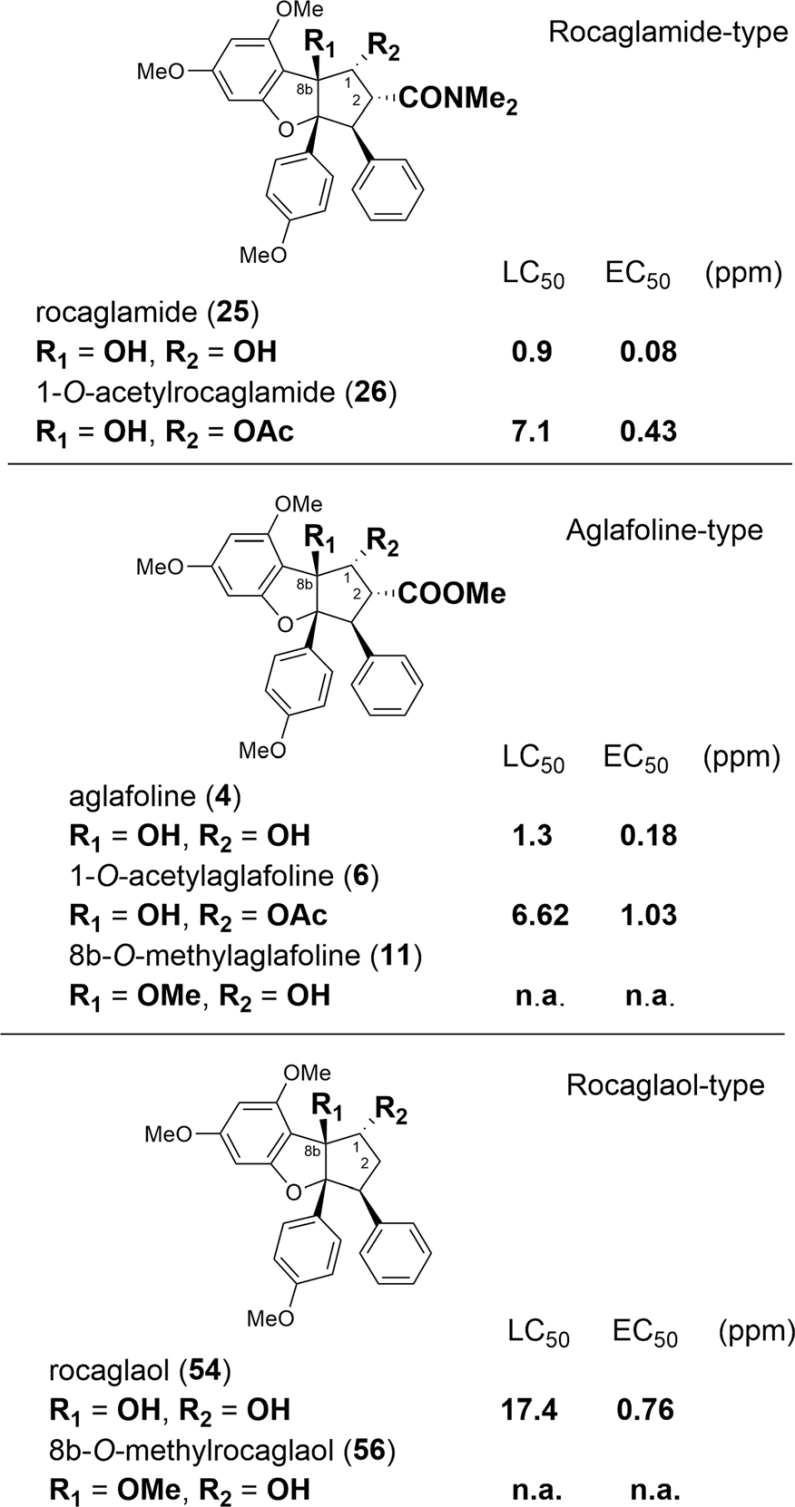
Insecticidal activities against *Spodoptera littoralis*

n.a. = not active up to a concentration of 100 ppm (Hiort et al. 1999; Chaidir et al. 1999)

### Antiproliferative activities

Inspired by the significant antileukemic activity of the alcoholic extract from stems and roots of *A. rimosa* (= *A. elliptifolia*), King et al. (1982) examined the chloroform-soluble fraction to identify the active principle. They isolated and identified the first benzofuran flavagline, named rocaglamide (**25**), which showed high activity against P388 lymphocytic leukemia. In spite of this pronounced activity further research on antitumor properties of flavaglines only started 14 years later, when Dumontet et al. (1996) reported on the strong cytotoxic activity of didesmethylrocaglamide (**38**) with an inhibitory concentration (IC_50_) value of 6.0 ng/ml with KB (oral epidermoid carcinoma) cells. Since then different research groups investigated the inhibitory effects of flavaglines in different tumor cells. In the following year Wu et al. (1997) reported on the high cytotoxicity of aglafoline (**4**) and rocaglamide (**25**), isolated from the stems of *A. rimosa*, against six cancer cell lines with IC_50_ values in the range of 1–7 ng/ml. Structure–activity relationships were observed in five benzofurans, isolated from the fruits and stems of *A. elliptica* Blume (Cui et al. 1997). Structurally, they were shown to represent aglafoline (**4**, **18**, **19**)- and rocaglaol-type derivatives (**61**, **63**). Apart from aglafoline (**4**) itself, they deviate from the parent compounds by a methylenedioxy group at *C*-3′–C-4′ of the B-ring, and different substituents at *C*-1. As shown in Table 6 all compounds were active in various human tumor cell lines, but the responses mediated by the three aglafoline-type derivatives **4**, **18**, and **19** were particularly potent with IC_50_ values in the range of 1–30 ng/ml. Here, 4′-demethoxy-3′,4′-methylenedioxyaglafoline (**18**) showed the highest cytotoxicity against glioblastoma (U373) and breast cancer (BC1) cell lines with IC_50_ values at 0.8 and 0.9 ng/ml, respectively, whereas aglafoline (**4**) itself showed lower values at 10.0 and 3.0 ng/ml. By contrast, the loss of a substituent at *C*-2 in the rocaglaol-type derivatives **61** and **63** clearly showed less activity. In BC1 cells compound **61** exhibited an IC_50_ value only at 200.0 ng/ml, while in **63** the replacement of the *OH*-group at *C*-1 by an oxo-group led to an even bigger reduction at 1400.0 ng/ml. It is interesting to note, that 3′,4′-methylenedioxy substitution in ring B led here to a more potent activity (Table 6), while the presence of a 3′-OH or 3′-OMe group in the derivatives **7**, **27**, **28**, **31**, **35**, **36**, **40** functions as a negative factor (Bohnenstengel et al. 1999a). The remarkable cytotoxicity of the aglafoline-type derivative **18** led to a mechanistic study of its activity and to in vivo testing of selected compounds. In accord with the in vitro results compound **18** inhibited the growth of BC1 cells implanted in athymic mice. As demonstrated with clonogenic and cell proliferation assays performed by treating lung cancer cells (Lu1) with compound **18**, growth inhibitory activity was reversible, and cells retained viability. These tests suggested that compound **18** functions by a cytostatic mechanism, probably due to inhibition of protein biosynthesis, rather than inducing necrosis or apoptosis (Lee et al. 1998).

**Table 6:**
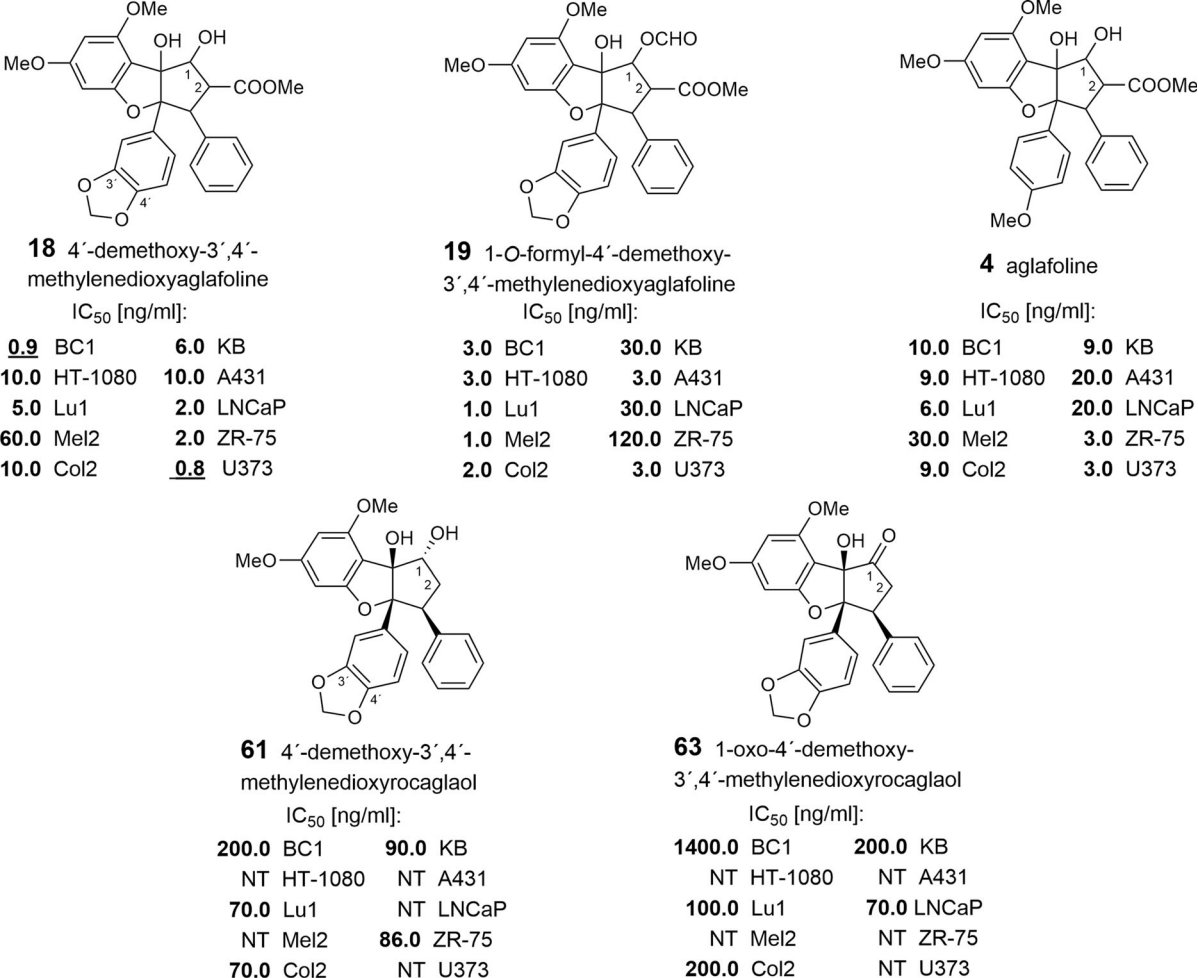
Growth inhibitory potential of five benzofuran flavaglines with various human cancer cell lines^a^

^a)^Key: BC1, Breast cancer; HT-1080, fibrosarcoma; Lu1, lung cancer; Mel2, Melonoma; Col2, colon cancer; KB, oral epidermoid carcinoma; A431, epidermoid carcinoma; LNCaP, hormon dependent prostate cancer; ZR-75, hormon dependent breast cancer; U373, glioblastoma; NT, not tested (Cui et al. 1997; Lee et al. 1998)

Structure–activity relationships were also shown by testing eleven benzofurans and one benzopyran for growth inhibiting properties against the two human cancer cell lines MONO-MAC-6 (monocytic leukemia) and MEL-JUSO (melanoma) (Bohnenstengel et al. 1999a). While the benzopyran aglain (**78**/**79**) was shown to be completely inactive, all of the benzofurans studied exhibited pronounced antiproliferative activity. The most active compound was didesmethylrocaglamide (**38**) with IC_50_ values at 2.0 ng/ml with MONO-MAC-6, and 6.0 ng/ml with MEL-JUSO, and is thus comparable with the wellknown anticancer drug vinblastine sulfate. A slight decrease of activity was observed by replacing the *CONH*_2_-group of **38** by larger substituents, and the acetylation of the *OH*-group at *C*-1. A negative influence was also observed by the insertion of *OH* or *O*Me at *C*-3′ of the B-ring (Bohnenstengel et al. 1999a). Further studies indicated a complete loss of antiproliferative activity by replacing the *OH* at *C*-8b by a methoxy group in compounds **11** and **56**. Comparing these findings with those reported for insecticidal activities (Table 5), it is conceivable to assume that the molecular mechanisms of flavaglines are similar or identical both in insects and human cancer cells (Bohnenstengel et al. 1999b).

From six flavaglines, isolated from the leaf extract of the Malaysian *A. laxiflora* Miq., only the two benzofurans, rocaglaol (**54**) and its 3′-rhamnoside (**60**), showed significant activity against a panel of cancer cell lines, while the four benzopyrans **90**, **94**, **95**, and **102** were inactive. This study reported for the first time on the cytotoxicity of a flavagline glycoside (Xu et al. 2000). The *C*-2 unsubstituted rocaglaol (**54**) was later also isolated as the main cytotoxic constituent from the bark of *A. crassinervia* Kurz ex Hiern. In contrast to previous results, where the loss of the carboxy group at *C*-2 led to a decrease of activity in **61** and **63** (Lee t al. 1998), the high ED_50_ values of rocaglaol (**54**) itself against Lu1 (lung cancer), LNCaP (hormone-dependent prostate cancer), and MCF-7 (breast cancer) were comparable to those of the positive controls, paclitaxel (Taxol®), and camptothecin. Moreover, compound **54** was found to be selectively (> 330-fold) active against the three cell lines, when compared with the non-tumorigenic HUVEC cell line (Su et al. 2006). Further experiments showed that it represents a potent cytotoxic agent that induces cell cycle arrest at the G_2_/M-phase and apoptosis through the mitochondrial pathway (Mi et al. 2006a). Cytotoxic activities against Lu1, LNCaP and MCF-7 cells in the range of ED_50_ values at 0.001 to 0.8 µg/ml were also reported for aglaroxin A (**47**), 1-*O*-acetylaglaroxin A (**48**), and 1-*O*-acetylaglaroxin B (**51**), all of which are characterized by a 8-methoxy-6, 7-methylenedioxy substitution of the aromatic ring A (Kim et al. 2006a).

Significant cytotoxicity was also determined for rocagloic acid (**1**) from the leaves of *A. rimosa*, representing a benzofuran with a free carboxy group at *C*-2 (Wang et al. 2001a, b). Compound **1** was later also isolated from the twigs of *A. rubiginosa* (Hiern) C. M. Pannell together with the *C*-2 methylesters aglafoline (**4**) and 1-*O*-acetylaglafoline (**6**), and the *C*-2 unsubstituted 1-*O*-acetylaglaol (**55**). All four compounds exhibited broad cytotoxic activity, with ED_50_ values in the range of 63–325 nmol against a panel of human cancer cell lines (Rivero-Cruz et al. 2004). Using a colorimetric method the cytotoxic properties of seven flavaglines, isolated from the fruits of *A. cucullata* (Roxb.) Pellegrin, were evaluated against KB (oral epidermoid carcinoma), BC (breast cancer), and NCI-H187 (small cell lung cancer) cell lines. In this assay, the rocagloic acid derivatives **2**, **3**, the aglafoline-type derivatives **4**, **5**, **7**, and rocaglaol (**54**) exhibited strong activities, whereas rocagloic acid (**1**) itself and 3′-hydroxyaglafoline (**7**) were found to be selectively toxic only against the NCI-H187 cell line (Chumkaew et al. 2006).

Another effect of functional group variation was observed by the fusion of a pyrimidinone moiety to *C*-2 and *C*-1. The cell growth inhibiting activity was first reported for aglaiastatin (**65**) and dehydroaglaiastatin (**66**) against Ki-ras-transformed NRK cells (Ohse et al. 1996), and later for **66** and aglaiformosanin (**68**) against five different cancer cell lines (Wang and Duh 2001). Analysis of the cytostatic effects and the underlying mechanisms of action of six benzofurans against the colorectal carcinoma cells SW480 and HT29/HI has shown, that all derivatives caused massive, concentration-dependent cell loss in both cell lines with the pyrimidinone aglaiastatin (**65**) as the strongest inhibitor. Compound **65** was found to induce growth inhibition by blocking cell cycle progression in the mitotic prophase and induce apoptosis. This study described for the first time the selective cytotoxicity of flavaglines, being highly specific for malignant cells, while normal and premalignant cells were shown to be 1.000-fold less sensitive (Hausott et al. 2004).

In a later investigation the *CHCl*_3_ extract of *A. perviridis* Hiern was found to exhibit high cytotoxic activity against human colon cancer (H-29) cells (Pan et al. 2013). From ten benzofuran flavaglines seven exhibited pronounced activity with ED_50_ values ranging from 0.0007 to 0.056 µmol with rocaglaol (**54**) as the most potent compound. In accord with previous findings (Bohnenstengel et al. 1999b), derivatives with the *C*-8b hydroxy group replaced by a methoxy group, including the newly described compounds **21** and **62**, were found to be much less potently cytotoxic. Interestingly, perviridisin B (**107**), a flavagline with a benzopyran structure, exhibited significant cytoxicity (ED_50_ 0.46 µmol) against HT-29 cells, while the *C*-10 epimer, perviridisin A (**106**), was inactive in the same assay. In order to evaluate the selectivity of these potent agents for a tumorigenic cell line, compounds with ED_50_ values of less than 10 µmol against HT-29 cells were further tested against the CCD-112CoN normal colon cell line. None of the compounds tested were found to show inhibitory activity against the normal cells at the relatively high concentration of 50 µmol (Pan et al. 2013). The important role of a free *OH* group at *C*-8b for cytotoxic activity was also confirmed in a phytochemical investigation of the twigs of *A. odorata* var. *microphyllina*. From five rocaglamide-type flavaglines tested against the human myeloid leukemia cell line (K562), only 3′-hyroxy-8b-methoxy-rocaglamide (**29**) and 8b-methoxy-desmethylrocaglamid (**34**) were inactive, while the other three derivatives showed pronounced cytotoxicity (Liu et al. 2013). A re-examination of the cytotoxic effects of rocaglaol (**54**) and rocaglamide (**25**) against five tumor cell lines exhibited IC_50_ values from 0.007 to 0.095 µmol with rocaglamide (**25**) as the most potent inhibitor (Liu and Xu 2016).

### Silvestrol as lead structure with high cytotoxic potency

An important structural modification with very high cytotoxic potency was found in silvestrol (**14**) and its 5′′′ S epimer episilvestrol (**15**), two benzofuran flavaglines with an unusual dioxanyloxy unit attached to *C*-6 of the aromatic ring A. They were isolated and identified from the fruits and twigs of *A. foveolata* C. M. Pannell (published as *A. silvestris* (M. Roemer) Merrill), and were found to possess comparable cytotoxic potencies for the cancer cell lines Lu1, LNCaP, and MCF-7 to those of the well-known natural anticancer compounds paclitaxel (Taxol) and camptothecin (Fig. 5). Silvestrol (**14**) was further evaluated as being active in vivo in the hollow fiber assay and in the murine P-388 leukemia model (Hwang et al. 2004). The evaluation of the mechanism of cytotoxicity in LNCaP cells has shown that the activity is associated with cell cycle arrest at the G_2_/M checkpoint and alterations in the expression of genes regulating apoptosis and cell cycle independent of tumor suppressor protein p53 activity (Mi et al. 2006b). A follow-up study demonstrated the involvement of the apoptosome/mitochondrial pathway and suggested the possibility that silvestrol (**14**) may also trigger the extrinsic pathway of programmed cell death signaling in tumor cells (Kim et al. 2007). Further experiments exhibited a dual targeting of compound **14** in mantle cell lymphoma with the cyclin/CDK/Rb pathway, a conserved mechanism controlling cell cycle progression, and the mitochondrial (intrinsic) pathway of apoptosis (Alinari et al. 2012). Silvestrol (**14**) also showed potential for the development as new therapeutic agent for B-cell malignancies including chronic lymphocytic leukemia (CLL). It exhibited a LC_50_ value of 7 nmol in CLL, being more potent against B-cells than T-cells, and significantly prolonged survival in a murine model of B cell acute lymphoblastic leukemia (ALL) (Lucas et al. 2009, 2010). However, a resistance to compound **14** was observed in ALL mediated by the multidrug-resistant (MDR1) gene, and its product P-gp (permeability glycoprotein) overexpression (Gupta et al. 2011). In another paper the authors described the pharmacokinetic properties of **14** in mice, using liquid chromatography/tandem mass spectroscopy (LC–MS/MS). They found that intraperitoneal systemic availability was 100%, but oral administration resulted in only 1.7% bioavailability (Saradhi et al. 2011). A synergistic effect of silvestrol (**14**) in combination with other therapeutic agents was reported for the inhibition of cell growth in four different hepatocellular cancer (Kogure et al. 2013). In acute myeloid leukemia (AML), one of the most common types of leukemia, silvestrol (**14**) was shown to exhibit significant in vivo and in vitro activities through a novel mechanism resulting in inhibition of *FLT3* (FMS-like tyrosine kinase receptor-3) and miR-155 (microRNA) expression (Alachkar et al. 2013). In in vitro and in vivo models of Epstein-Barr virus-driven lymphoproliferative diseases (EBV-LPD) silvestrol was shown to promote anti-tumor activity and simultaneously preserve the anti-tumor function of innate immune effectors (Patton et al. 2014). A study of Callahan et al. (2014) demonstrated that rocaglamide (**25**) and silvestrol (**14**) are able to preferentially kill functionally defined leukemia stem cells, while sparing normal stem and progenitor cells. In addition to efficacy as single agents, both flavaglines were shown to sensitize leukemia cells to several anticancer compounds. Compound **14** was also reported to potently inhibit growth of melanoma cells and induce cell death through induction of early autophagy and caspase-mediated apoptosis (Chen et al. 2016) (Fig. 5). Synergistic effects of compounds **14** and **15** in combination with cisplatin were observed in the inhibition of nasopharyngeal carcinoma cell proliferation (Daker et al. 2016). Recently, both silvestrols (**14**, **15**) were also isolated from the stems of *A. stellatopilosa* Pannell and their strong cytotoxicity was confirmed against the cancer cell lines H-29, MCF-7, and NCI-H460 (Othman et al. 2016).

### Structural variation of silvestrol

Interestingly, epimerization in the dioxanyloxy group of the silvestrols **14** and **15** leads to a dramatic decrease of cytotoxicity evaluated against the HT-29 human colon cancer cell line. While compounds **14** and **15** exhibited very high ED_50_ values of 0.0007 and 0.001 µmol, respectively, the two C-2′′′ epimers, 2′′′-episilvestrol (**16a**) and 2′′′, 5′′′-diepisilvestrol (**16b**), were found to be much less active, with 2.29 and 1.07 µmol (Pan et al. 2010). On the other hand, acetylation of the *OH* group at *C*-6′′′ of the dioxanyloxy unit in aglapervirisin A (**17**) retained significant cytotoxicity in four human tumor cell lines, with IC_50_ values between 0.008 and 0.015 µmol. Compound **17** induced cell cycle arrest in the G2/M-phase in Hep G2 (liver cancer) cells at concentration of 0.010 µmol, and induced apoptosis at 0.160 µmol (An et al. 2016).

In continuous efforts to discover new silvestrol derivatives with antineoplastic activity six new analogues (**41**–**46**) were recently isolated from the root extract of *A. perviridis* Hiern, where in all compounds the ester group at *C*-2 is replaced by a *CONH*_2_ substitiuent. Apart from this amide group, two derivatives (**41**, **42)** differ from silvestrol (**14**) by a free *OH* group at *C*-2′′′of the dioxanyloxy unit, while the four other derivatives (**43**–**46)** deviate by an unprecedented carbon skeleton with a dihydrofuran ring fused to the dioxanyloxy and the aromatic ring A. All compounds were evaluated for their cytotoxic activity against the HT-29 human colon cancer and PC-3 prostate cancer cell lines. The latter four compounds (**43**–**46**) were shown to be inactive (IC_50_ > 10 µmol) probably caused by the rigidity of the dioxanyl ring due to the fused dihydrobenzofuran ring system. The moderate activity of compound **41** (IC_50_ > 2.3 µmol) compared to silvestrol (**14**) could be ascribed to either or both the *OH* group at *C*-2′′′ or the amide group at *C*-2 (Agarwal et al. 2019).

### Inhibition of the translation initiation factor (eIF) 4A

**Fig. 6:**
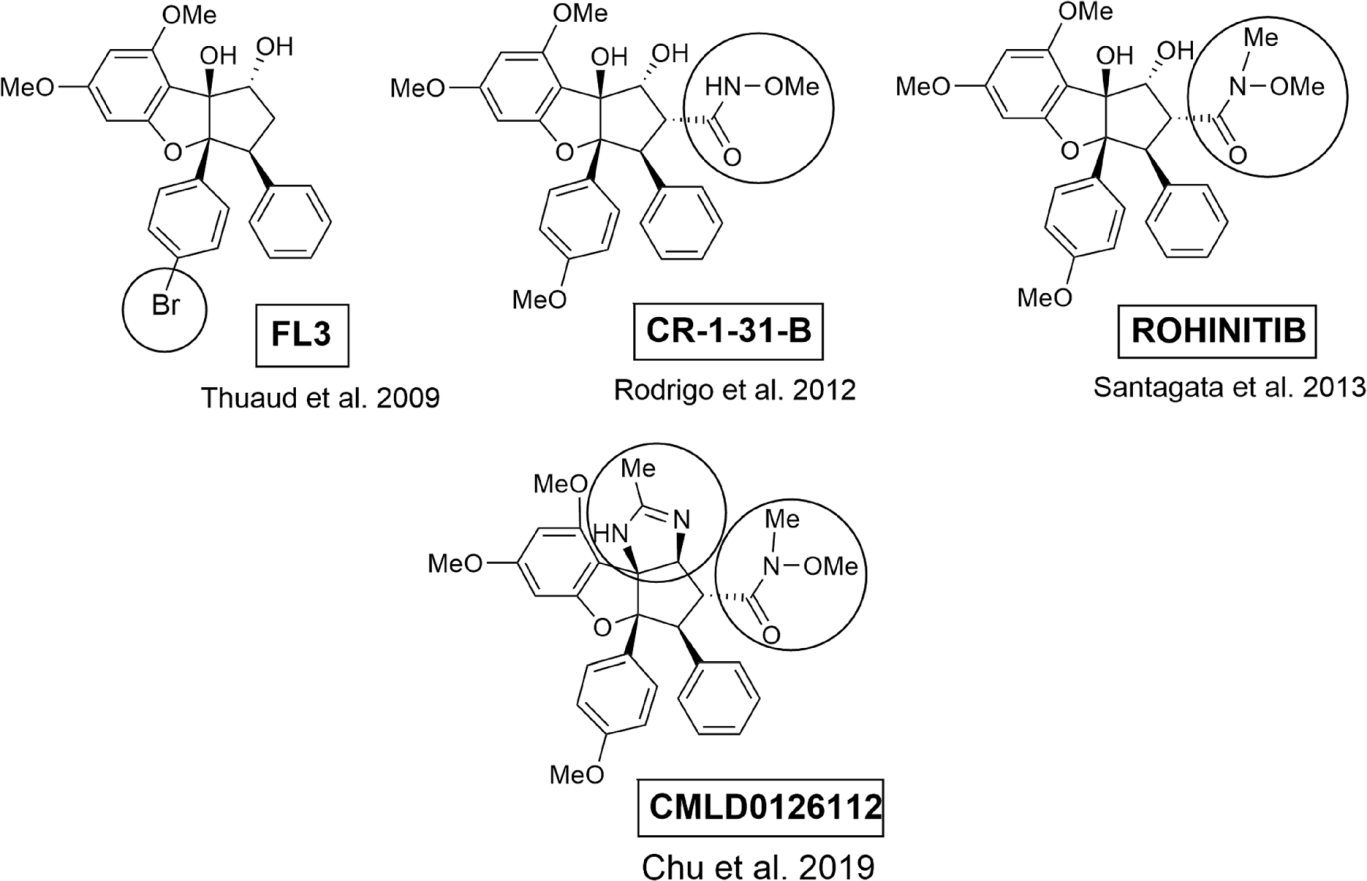
Synthetic flavaglines with high anti-proliferative activity. Encircled substituents indicate structural differences to the corresponding naturally occurring parent compounds rocaglaol (**54**) and rocaglamide (**25**)

The level of interest in the therapeutic potential of flavaglines has been intensified by uncovering their role as direct inhibitors of translation initiation, a key process in protein synthesis (Silvera et al. 2010; Schatz et al. 2011; Bhat et al. 2015; Pelletier et al. 2015; Chu and Pelletier 2015; Chu et al. 2016a; Taylor et al. 2020; Hao et al. 2020). Pelletier's group first demonstrated that the eukaryotic initiation factor (eIF) 4A was the likely target of flavaglines. The helicase eIF4A is known to unwind secondary structures at the 5′-UTRs (untranslated regions) of mRNA to enable binding of the ribosomal pre-initiation complex. The authors used biochemical assays to show that 1-*O*-formylaglafoline (**5**) and silvestrol (**14**) stimulated an abnormal interaction of eIF4A with mRNA and prevented successful assembly of the mRNA/eIF4A dimer with the eIF4F translaton initiation complex. It was also shown that silvestrol can re-sensitize tumor cells to standard-of-care agents, such as doxorubicin, in a lymphoma model (Bordeleau et al. 2008). In following experiments it was shown that targeting the eIF4A subunit of eIF4F in human cancer xenograft tissues is sufficient to reduce tumor cell proliferation and that silvestrol (**14**) was the most active derivative from twelve benzofurans tested. The authors provided a mechanistic insight into the mode of action of compound **14** and indicated that it is a potent anticancer compound in vivo by affecting survival pathways as well as angiogenesis and appears to be well tolerated in animals (Cencic et al. 2009, 2010). To determine unequivocally the protein targets of silvestrol (**14**) and biotinylated episilvestrol (**15**) Rizzacasa's group demonstrated for the first time their direct interaction with eIF4AI and eIF4AII (Chambers et al. 2013). In a second publication they showed that aglafoline (**4**) and rocaglamide (**25**) also bind specifically to eIF4AI/II but not to prohibitins (PHB), the helicase DDX3 and eIF4E (Chambers et al. 2016). This discovery was substantiated by chemogenomic profiling to validate eIF4A as the main target of flavaglines in yeast. Here, the binding site of flavaglines could be identified by means of mutagenesis and in silico modeling (Sadlish et al. 2013). Using a series of biochemical assays and CRISPR (clustered regularly interspaced short palindromic repeats)/Cas9 genome editing, Chu et al. (2016b) provided genetic evidence that the antineoplastic activities of flavaglines are a consequence of eIF4A1 inhibition. Iwasaki et al. (2016) have shown that rocaglamide (**25**) induces ATP-independent “clamping” of eIF4A onto polypurine sequences in the 5′-cap-UTR of mRNAs, creating an inihibitory roadblock for the scanning ribosome. Structural elucidation of a rocaglamide (**25**)/eIF4A1/polypurine RNA complex revealed that **25** functions as interfacial inhibitor and makes critical contacts with eIF4A1 (F163, Q195) and two adjacent RNA purine bases (Iwasaki et al. 2019). In a more recent investigation Iwasaki’s group found that the translation inhibitor rocaglamide (**25**) alternatively targets the helicase DDX3 in addition to eIF4A (Chen et al. 2021). This is contrary to the results of Chambers et al. (2016). Inhibition of eIF4A by silvestrol (**14**) was recently also shown to sensitize T-47D breast cancer cells to radiotherapy with minimal effects on unirradiated cells (Webb et al. 2020). In a screen of > 200 flavaglines (= rocaglates) a series of potent synthetic amidino-flavaglines was identified (e.g. CMLD012612, Fig. 6). They were shown to target eIF4A1 and eIF4A2, to potently inhibit translation and tumor cell viability, and are effective in synergizing with DNA-damaging agents in vivo against the MYC (proto-oncogene)-driven lymphomas (Chu et al. 2019). Despite showing promising anticancer activities, the development of flavagline derivatives as therapeutic agents has been hampered because of poor drug-like properties and synthetic complexity. Utilizing a ligand-based design strategy to optimize physicochemical properties and mechanistic studies to further elucidate mRNA sequence selectivity, key regulated target genes, and the associated antitumor phenotype, the synthetic flavagline eFT226 was designed. This compound, also named zotatifin, showed excellent physicochemical properties and significant antitumor activity and is already under clinical investigation (Ernst et al. 2020).

### Prohibitins (PHB)-1 and 2 as targets of flavaglines

Two conflicting reports have been published concerning the protein targets. Li-Weber and colleagues at the German Cancer Research Center (DKFZ) have demonstrated that several benzofuran flavaglines prevent tumor growth and sensitize resistant cancer cells to apoptosis by blocking the Raf-MEK (mitogen and extracellular-signal regulated protein kinase)-ERK (extracellular-signal regulated protein kinase) signaling pathway. However, the molecular target of flavaglines in this pathway remained unknown (Zhu et al. 2007; Bleumink et al. 2011). In a following study, using an affinity chromatography approach, it was found that prohibitins (PHB)-1 and 2 are the direct targets of flavaglines (Polier et al. 2012) and it was therefore concluded that flavagline binding to PHB prevented it from interaction with cRaf, and thus inhibiting translation indirectly by blocking the Raf-MEK-ERG pathway. However, in accord with the results of Chambers et al. (2016), previous work suggested that PHB 1/2 are not likely to be involved in the mode of action of translation inhibition as flavaglines have retained translational activity in cellular extracts, which do not have a functional Raf-MEK-ERG pathway, which is responsible for communication between extracellular receptor and the nucleus (Bordeleau et al. 2008). The synthetic flavagline FL3 (Fig. 6), was shown to directly target PHB and inhibits UCB (urothelial carcinoma of the bladder) cell proliferation both in vitro and in vivo (Yuan et al. 2018). As PHB proteins are involved in regulation of several important signaling pathways in different cellular compartments (Thuaud et al. 2013), it was suggested that flavaglines may serve as a new small-molecular tool for studying PHB-mediated cellular processes (Bentayeb et al. 2019). In a more recent study PHB2 was identified to mediate mitophagy, a conserved cellular process for selectively removing damaged or unwanted mitochondria. It was found that the synthetic flavagline FL3 inhibits PHB2-mediated mitophagy and blocks cancer cell growth at nanomolar cocentrations (Yan et al. 2020).

### Targeting the transcriptional factor HSF1

The transcriptional factor HSF1 (heat shock factor 1) is deeply involved in metabolic programming, survival, and proliferation of cancer cells in addition to heat-shock response. To shed light on how the inhibition of protein synthesis selectively impedes the proliferation of cancer cells without effecting normal cells more than 300,000 compounds were screened for the inhibition of the transcriptional factor HSF1. Among these compounds rocaglamide (**25**) was found to be the most potent and selective inhibitor of HSF1 signaling (IC_50_ ~ 50 nmol). Five synthetic hydroxam analogues showed even more activity with rohinitib (Fig. 6) as the strongest inhibitor (IC_50_ ~ 20 nmol) (Santagata et al. 2013).

### Antiviral activities

Since the replication of several viruses relies on the host translation system, a dependence on the initiation factor eIF4A for their translation by the host protein synthesis was to be expected. Inspired by the role of flavaglines as direct inhibitors of translation initiation Grünweller and colleagues investigated the inhibiting effect of silvestrol (**14**) on the replication of Ebola virus (EBOV), a negative-stranded RNA virus. Silvestrol was known to exert potent antitumor activity by inhibiting translation of short-lived onco-proteins (Lucas et al. 2009; Kogure et al. 2013), whose mRNA contains extended 5′-cap-UTRs (untranslated regions) and includes regions of stable RNA secondary structures that require unwinding by the helicase eIF4A. Since the mRNAs of EBOV adopt stable RNA secondary structures in their UTRs, a corresponding mechanism was assumed. The authors observed that silvestrol (**14**) inhibited an infection of EBOV in human macrophages at low nanomolar concentrations, and effective silvestrol concentrations were non-toxic in the tested cell systems (Biedenkopf et al. 2017). Many plus-stranded RNA viruses, such as corona virus (CoV) and the picornaviruses human rhinovirus A1 (HRV A1) and poliovirus typ1 (PV), carry long and highly structured 5′-cap-UTRs with important functions in viral replication and/or translation initiation. Using a dual luciferase assay and virus-infected primary cells, Müller et al. (2018) found that silvestrol (**14**) is also a potent inhibitor of cap-dependent viral mRNA translation in CoV-infected human embryonic lung fibroblast (MRC-5) cells. High EC_50_ values at 1.3 nmol and 3.0 nmol silvestrol were determined for MERS-CoV (Middle East Respiratory Syndrome coronavirus) and HCoV-229E (human coronavirus 229E), respectively. For the highly pathogenic MERS-CoV, the potent antiviral activity of compound **14** was alo confirmed using peripheral blood mononuclear cells as a second type of human primary cells. It was shown that silvestrol strongly inhibits the expression of CoV structural and nonstructural proteins and the formation of viral replication/transcription complexes. Furthermore, antiviral effects of **14** against HRV A1 and PV were also investigated. Both viruses employ an Internal Ribosomal Entry Site (IRES)-mediated translation initiation mechanism. For PV, which is known to require the activity of eIF4A, an EC_50_ value at 20 nmol silvestrol (**14**) was determined in MRC-5 cells. The higher value at 100 nmol measured for HRV A1 indicated a less critical role of eIF4A activity in HRV A1 IRES-mediated translation initiation (Müller et al. 2018).

A series of following investigations confirmed the antiviral properties of flavaglines and contributed to a deeper understanding of the mode of action. Silvestrol (**14**) was also shown to inhibit the replication of HEV (hepatitis E virus), and was evaluated for its specific inhibition of eIF4A in different HEV experimental model systems including HEV primary isolates and HEV-infected humanized mice. It was demonstrated that silvestrol blocks replication of different subgenomic replicons in a dose-dependent manner at low nanomolar concentrations. Moreover, a combined treatment of viral infection with ribavirin, a nucleosid inhibitor, revealed an additive effect (Todt et al. 2018). In another study the impact of silvestrol (**14**) on the HEV life cycle was analyzed. It was found that **14** is a potent inhibitor of the release of HEV infectious viral particles, and that this effect goes along with a strongly reduced HEV capsid protein translation, retention of viral RNA inside the cytoplasm, and without major cytotoxicity (Glitscher et al. 2018). Silvestrol (**14**) was also active against two strains of ZIKV (Zika virus) using lung adenocarcinoma (A549) cells and primary human hepatocytes (PHH). However, the results showed that the silvestrol-dependent effects on the ZIKV life cycle did not follow a strict dose–effect relation in A549 cells. While 5 nmol and 50 nmol silvestrol exerted a strong inhibition of ZIKV replication, 10 nmol failed to impair replication most likely due to secondary effects in the infected host cell (Elgner et al. 2018). The antiviral activity of silvestrol (**14**) was also reported for CHIKV (chikungunya virus), a positive single-stranded RNA virus (Henss et al. 2018). Interesting results were obtained by comparing the broad-spectrum antiviral activities of the eIF4A inhibitor silvestrol with those of the synthetic amidino-flavagline CR-1-31-B (Fig. 6), lacking the dioxane moiety of silvestrol. Even though similar antiviral activities were found for this less complex structured flavagline in different viruses, substantial mechanistic differences were observed. RNA clamping with CR-1-131-B depends on a polypurine stretch in the bound RNA substrate whereas silvestrol (**14**) with its dioxanyloxy moiety can also clamp RNA onto eIF4A without a strict requirement for a polypurine stretch in the RNA substrate (Müller et al. 2020). In a following paper Müller et al. (2021) assessed the antiviral activity of CR-1-131-B against SARS-CoV-2 using both in vitro and ex vivo cell culture models. In Vero E6 cells it inhibited SARS-CoV-2 replication with an EC_50_ of ~ 1.8 nmol. In primary human airway epithelial cells CR-1-131-B reduced viral titers to undetectable levels at a concentration of 100 nmol. Reduced virus reproduction was accompanied by substantially reduced viral protein accumulation and replication/transcription complex formation. Taken together, the data available so far characterize the complex antiviral activity of flavaglins and show their broad spectrum of functions (Fig. 5). As recently highlighted by Schulz et al. (2021) flavaglines are not only promising anticancer agents but gained now also high expectations as agents against emerging RNA viruses like SARS-CoV-2. This was outlined in a more recent review by Taroncher-Oldenburg et al. (2021), where they described the targeting of the helicase eIF4A with flavaglines as a pan-antiviral strategy for minimizing the impact of future RNA virus pandemics.

### Antiprotozoal activities

In a preliminary screening for biologically active constituents the ethanol extract of the seeds of *A.erythrosperma* C. M. Pannell exhibited significant antimalarial activity. In a microculture radioisotope assay against a multidrug resistant strain (K1) of the malarial parasite *Plasmodium falciparum*, the benzofuran flavagline 4′-demethoxy-3′,4′-methylenedioxyaglafoline (**18**) was shown to be responsible for the anti-plasmodial activity with an IC_50_ value of 7.30 µg/ml (Phongmaykin et al. 2011). In the course of investigating the anti-leishmanial activity of 13 plant-derived compounds against *Leishmania infantum* promastigotes in vitro, the benzofurans aglafoline (**4**) and rocaglamide (**25**) showed promising potencies with 50% effective concentrations (EC_50_) of 7.45 and 16.45 µmol, respectively, after 24 h of exposure (Astelbauer et al. 2011). In a follow-up study these two flavaglines were also tested for their anti-plasmodial activity in vitro. In this case, fresh *Plasmodium falciparum* isolates were taken from patients in the area of Mae Sot in NW Thailand and the inhibition of schizont maturation was determined. With IC_50_ values of 53.49 nmol for aglafoline (**4**) and 60.59 nmol for rocaglamide (**25**) the activities were significantly below that of artemisinin, but moderately higher than that of quinine (Astelbauer et al. 2012). In continuation of this screening program for antiprotozoal activities aglafoline (**4**) also showed pronounced activity against *Giardia duodenalis* trophozoites colonizing the small intestine. The EC_50_ values after 24 h and 48 h were 17.2 µmol and 7.71 µmol, respectively (Drinić et al. 2019).

In the treatment of malaria recent studies have pointed to initiation of protein synthesis as a novel pharmacological target. Considering the role of flavaglines as inhibitors of protein synthesis by targeting the translation initiation factor eIF4A, the synthetic flavagline CR-1-31-B (Fig. 6) was tested for inhibitory effects in *Plasmodium* parasites. It was found that this compound perturbs association of *Plasmodium falciparum* eIF4A (PfeIF4A) with RNA. It showed potent prophylactic and therapeutic antiplasmodial activity in vivo in mouse models of infection with *P. berghei* (cerebral malaria) and *P. chabaudi* (blood-stage malaria) and can also block replication of different clinical isolates of *P. falciparum* in human erythrocytes, including drug-resistant isolates (Langlais et al. 2018).

### Anti-inflammatory activities

The anti-inflammatory properties of crude extracts of different *Aglaia* species were supposed to be mediated by the transcription factor NF-κB, which plays a key role in regulating the immune response to infection. Baumann et al. (2002) tested sixteen benzofuran flavaglines and one benzopyran flavagline for their ability to inhibit NF-κB activity. The three benzofuranes didesmethylrocaglamide (**38**), desmethylrocaglamide (**32**) and rocaglamide (**25**) showed a dose-dependent inhibition of PMA (phorbol myristate acetate)- and TNF (tumor necrosis factor)-induced NF-κB activation in Jurkat T cells, and mediated an almost complete inhibition at a final concentration of 200 nmol. Compound **38** was found to be the most active NF-κB-specific derivative with IC_50_ values at 58 nmol for TNF- and 44 nmol for PMA-induced activity. A replacement of the amide group at *C*-2 by *COO*Me or by *C*-2 unsubstituted derivatives as well as the insertion of a hydroxy or methoxy group in *C*-3′ led to a reduction of NF-κB inhibitory potential. A decrease of activity was also observed by an introduction of either a 3′,4′- or 6,7-methylenedioxy group. However, the most dramatic loss in NF-κB inhibition was observed by replacing the *OH* group at *C*-8b by a methoxy group. Also, the replacement of the benzofuran backbone by a benzopyran skeleton led to a total loss of activity (Baumann et al. 2002). It is interesting to note that these results correspond well with those of findings mentioned above, where insecticidal properties and antiproliferative activities were determined (Bohnenstengel et al. 1999b). In a search for anticancer agents ten benzofuran flavaglines, isolated from *A. perviridis*, were also evaluated for their NF-κB (p65) inhibitory activity. With an ED_50_ value of 0.005 µmol rocaglaol (**54**) was extremly active, being 10 times more potent than the control compound rocaglamide (**25**) (Pan et al. 2013).

Li-Weber and colleagues showed that flavaglines are potent immunosuppressive compounds that suppress the production of several cytokines in peripheral blood T-cells at nanomolar concentrations. They also found that the doses that inhibit cytokine production, selectively inhibit the activity of NF-AT (Nulclear Factor of Activated T-cells) without impairing the activities of the transcription factors NF-κB and AP-1 (Activator Protein-1) (Proksch et al. 2005). A synthetic monofluor derivative of rocaglaol (**54**) displayed potent anti-inflammatory properties in human endothelial and murine glial cells in vitro. It was identified as a potent inhibitor of cytokine-mediated signaling and showed neuroprotective activity in vitro and in animal models of Parkinson ‘s disease and traumatic brain injury (Fahrig et al. 2005). In an enzyme-based ELISA assay the newly described benzopyran flavagline ponapensin (**133**), isolated from the leaves and stems of *A. mariannensis* Merrill (= *A. ponapensis* Kanehira), was shown to exhibit significant NF-κB inhibitory activity. With an IC_50_ value of 0.06 µmol it was more potent than the two benzofuran flavaglines rocaglamide (**25**) and aglafoline (**4**), with values of 2.0 and 2.3 µmol. The other benzopyrans (**78**, **79**, **80**, **85**), isolated in this study, were not active (IC_50_ > 5 µmol) (Salim et al. 2007b). In a more recent study the immunomodulatory effects of silvestrol (**14**) on human monocyte-derived macrophages and dendritic cells was investigated. Compound **14** was shown to down-regulate several pro- and anti-inflammatory cytokines and increased TNF-α during differentiation and activation of M1-macrophages, suggesting that the effects of silvestrol might cancel each other out. However, silvestrol (**14**) amplified the anti-inflammatory potential of M2-macrophages by increasing expression of anti-inflammatory surface markers (CD206, TREM2) and reducing release of pro-inflammatory cytokines (IL8, CCL2). The results showed that silvestrol (**14**) influences the inflammatory status of immune cells depending on the cell type and activation status (Blum et al. 2020).

### Antifungal activities

In a routine screening for antifungal properties the dichloromethane extract of the stem bark of *A. elaeagnoidea* showed activity against *Cladosporium cucumerinum* in a TLC bioassay. Activity-guided fractionation led to the isolation of aglafoline (**4**) which inhibited the growth of the fungus at 2.5 µg (Fuzzati et al. 1996). Using the spore germination inhibition assay in microwells, eight flavaglines, isolated from *A.odorata*, *A. elaeagnoidea* and *A.edulis*, were tested against the three plant pathogenic fungi, *Pyricularia grisea*, *Alternaria citri*, and *Fusarium avenaceum*. Based on digital image analysis of germ tubes, six benzofuran flavaglines showed clear antifungal activities, whereas a benzopyran and a benzoxepine flavagline were inactive. High activities were observed especially against *Pyricularia grisea*, the causative fungus of rice blast disease, with MIC values of 1.6 µg/ml for rocaglaol (**54**), 3 µg/ml for aglafoline (**4**), and 25 µg/ml for rocaglamide (**25**). Lower values were determined for pannellin (**22**) with 50 µg/ml, and for aglaroxin A (**47**) and desmethylrocaglamide (**32**) with 100 µg/ml each (Engelmeier et al. 2000). The lipophilic crude extracts from different *Aglaia* species were also tested for their effectiveness of growth inhibition against postharvest pathogenes. Bioassay-guided fractionation led to the isolation of the benzofuran flavaglines aglafoline (**4**), didesmethylrocaglamide (**38**), and rocaglaol (**54**), which exhibited significant antifungal activities against three different microfungi. The highest activities were determined for rocaglaol (**54**) with an EC_50_ value at 0.05 μg/ml against *Pestalotiopsis* sp., followed by 1.2 μg/ml against *Botrytis cinerea*, and 52 μg/ml against *Colletrotrichum gloeosporioides* (Khewkhom et al. 2006). Silvestrol (**14**) and episilvestrol (**15**) exhibited growth inhibition activity of > 90% against the two yeasts *Saccharomyces cerevisiae* and *Rhodoturula glutinis*, at a concentration of 200 µmol (Othman et al. 2016). In a more recent investigation it was found that a group of synthetic flavaglines activates a species-specific cell death program by translation inhibition in the emerging fungal pathogen *Candida auris* (Iyer et al. 2020).

### Miscellaneous properties

The platelet-activating factor (PAF), a phospholipid mediator, plays an important role in inflammatory response and mediation. In the search for specific PAF antagonists Ko et al. (1992) found that aglafoline (**4**), isolated from *A. rimosa*, selectively inhibited PAF-induced platelet aggregation in washed rabbit platelets in a concentration-dependent manner. In a following study the coexisting rocaglamide (**25**) was also tested for inhibition of platelet aggregation induced by adenoside diphosphate (ADP), arachidonic acid (AA), PAF, and collagen. It was shown that 100 µg/ml of aglafoline (**4**) completely blocked the platelet aggregation caused by PAF and AA but had no effect on that caused by ADP or collagen, while rocaglamide (**25**) were either completely inactive or showed only slight inhibition at the doses tested (Wu et al. 1997).

The leaf extract of *A. forbesii* was tested for anti-mycobacterial activity. From three benzopyran flavaglines isolated, isofoveoglin (= desacetylpyramidaglain D) (**111**) exhibited the highest activity against *Mycobacterium tuberculosis* with a MIC-value at 25 µg/ml which was compared with the two positive controls kanamycin with 1.25 µg/ml and isoniazid with 0.25 µg/ml (Joycharat et al. 2008a). Moderate anti-mycobacterial activity with a MIC-value at 50 µg/ml was also reported for the benzofuran 4′-demethoxy-3′,4′-methylenedioxyaglafoline (**18**) from the seed extract of *A. erythrosperma*. The activity was assayed in duplicate, using the Alamar blue test, and MIC-values below 200 µg/ml were considered active (Phongmaykin et al. 2011).

## Chemotaxonomic significance

The formation of flavaglines represents a unique chemical character of the genus *Aglaia* comprising around 120 species. Together with the characteristic indumentum of stellate hairs or peltate scales it distinguishes *Aglaia* from the other genera in the family Meliaceae. However, as some species are very similar in their morphology, taxonomic identification of the genus to the level of the species turned out to be difficult. Even the most indicative morphological characters vary considerably, so that Pannell (1992, 1994) adopted a wide species concept. Mainly based on fruit characters the genus is divided into the section *Amoora* with dehiscent, and section *Aglaia* with indehiscent fruits. The third section *Neoaglaia* was later proposed for species with dehiscent fruits but with flower characters intermediate between *Amoora* and *Aglaia*. A broad-based UV-HPLC comparison of crude extracts of 30 different *Aglaia* species has shown that flavaglines are widespread in both sections *Aglaia* and *Amoora*, but could not be detected so far in different accessions of *A. lawii* (Wight) Saldanha and *A. teysmanniana* (Miq.) Miq., mainly constituting the section *Neoaglaia*. Preliminary work combining into a systematic framework data from morphological characters, DNA analysis and phytochemicals has already been published (Muellner et al. 2005, 2009).

The UV-HPLC profiles of crude extracts of the widespread and morphologically variable *A. elaeagnoidea* (A. Juss.) Benth. were compared in our laboratory to assess the wide species concept proposed by Pannell (1992). Identification of the major flavaglines revealed a dominating 8-methoxy-6,7-methylenedioxy substitution in ring A. This biogenetic trend was also found in a large-scale collection (500 kg) of the stem bark of *A. elaeagnoidea* (syn.: *A. roxburghiana*), collected in Sri Lanka, where it was detected in a series of derivatives with the two benzofurans aglaroxin A (**47**) and aglaroxin C (**72**) as major constituents (Molleyres et al. 1999). The same substructure of ring A was found to prevail in different geographical provenances of *A. elaeagnoidea* from Bangladesh, Thailand, and Vietnam (Hofer 2002; Muellner et al. 2009), where it was also detected in the co-ocurring benzopyrans, benzoxepines, and flavonoids, as well as in the structurally related aglalactone (Brader et al. 1998; Bacher et al. 1999; Seger et al. 2000). In contrast, the HPLC profiles of *A. elaeagnoidea* collected in Australia clearly differ by flavaglines with the widespread 6,8-dimethoxylation of ring A. Based on chromatographic comparison with authentic samples the major compounds were identified as the benzofurans rocaglamide (**25**) and aglafoline (**4**) (Hofer 2002; Brem 2002). This deviating chemical profile together with differences in leaf morphology, fruit colour, and DNA data (Muellner et al. 2009), suggests a taxonomic rearrangement of the Australian accessions as a separate species.

The 8-methoxy-6,7-methylenedioxy substitution of flavaglines was also found to be typical for different collections of *A*. *edulis* (Roxb.) Wall., with aglaroxin A (**47**) as major compound. As shown in the stembark and roots of a 20 m tall tree, collected in southwest Thailand, compound **47** was accompanied by pannellin (**22**) and a series of benzopyrans (**123**, **129**–**133**) and benzoxepines (**143**, **145**), all of them were characterized by the same substitution pattern of ring A (Bacher et al. 1999). This common trend was also detected in an accession from Indonesia, where in addition to aglaroxin A (**47**), the closely related derivatives **48** and **51** were isolated, together with the benzopyrans **124**–**128**, and the benzoxepines **146**–**148** (Kim et al. 2005, 2006a). All these findings were in contrast to a previous report on *A. edulis* from southeast Thailand, where no flavaglines could be detected at all. In this case, a unique threefold acylated putrescine derived imide, edulimide (Table 2B), was isolated as the main component (Brader et al. 1998). This suggested a taxonomic segregation from *A. edulis*, also supported by a different type of indumentum showing peltate scales, instead of pale brown stellate hairs typical for *A. edulis*. However, according to Pannell (1992), *A. edulis* comprises 35 synonyms which cannot conveniently be subdivided into separate species or subspecies on present morphological characters. Since intermediate representatives are to be expected which have both hairs and scales, only further collections with flowers and fruits could come up to a final taxonomic decision. The taxonomic separation of *A. edulis* from *A. elaeagnoidea* is based on its larger leaves and much larger (edible) fruits (Pannell 1992), and is also suggested by DNA data. However, due to the variability of the leaf size, large leaved specimens from *A. elaeagnoidea* can sometimes be almost indistinguishable from *A. edulis* (Muellner et al. 2009).

Regarding the chemotaxonomic significance of the 8-methoxy-6,7-methylenedioxy substructure for *A. elaeagnoidea* and *A. edulis*, its formation in *A. oligophylla* Miq., collected in Vietnam (Dreyer et al. 2001; Bringmann et al. 2003), appears unusual. Even more, as other accessions of *A. oligophylla*, collected in Thailand, can be clearly distiguished by the constant predominance of rocaglaol (**54**), which structurally differs by the more widespread 6,8-dimethoxylation of ring A (Brem 2002). This predominant trend towards rocaglaol (**54**) in *A. oligophylla* was later also confirmed for another collection from south Thailand (Joycharat et al. 2008b). With respect to 6,8-dimethoxylation of the dominating flavaglines **66**, **67**, and **78,** the Chinese *A. testicularis* C.Y. Wu (Wang et al. 2004a) can be chemically distinguished from *A. edulis*, where it is currently treated as a synonym (Pannell 1992).

Based on different UV-HPLC profiles of the crude extracts from the widespread *A. tomentosa* Teijsm. & Binn., collected in Thailand, Malaysia, and Australia, the accessions can be clearly separated into two groups. One group is characterized by the genus-specific flavagline/bisamide profile, while the other differs by dominating lignans (Bachratá 2008). Identification of the major components revealed a predominance of rocaglaol (**54**) in all flavagline containing accessions (Brem 2002), while the lignan producing group is characterized by the dominating ( +)-methylarctigenin, a dibenzylbutyrolactone-type lignan (Brader et al. 1998; Bachratá 2008). The Australian accessions of *A. tomentosa* were exclusively characterized by flavaglines, showing a nearly identical HPLC profile to those collected in Thailand. Apart from the dominating rocaglaol (**54**), already published for its synonym *A. ferruginea* C.T. White & Francis (Mulholland and Naidoo 1998), the profiles consisted of small amounts of non-identified flavaglines, probably pyrimidinones and benzopyrans, and bisamides (Bachratá 2008). In contrast to this uniform and widespread profile detected in Thailand and Australia, the lignan-containing accessions from Thailand indicated affinities to *A. cordata* Hiern, where structurally similar aryltetrahydronaphthalene lignans were isolated (Wang et al. 2001b, 2002, 2004b). These findings would suggest a segregation of *A. cordata* from *A. tomentosa*, where it is currently treated either as a synonym (Pannell 1992) or subspecies (Pannell 1994). Regarding the chemical affinity of *A. tomentosa* to other members of the *A. tomentosa* group, comprising 13 species, *A. exstipulata* (Griffith) Theobald appears to be the nearest relative. Many accessions of this species collected in Thailand exhibited a constant flavagline pattern with rocaglaol (**54**) as dominating compound. By contrast, *A. archboldiana* A.C. Smith and *A. tenuicaulis* Hiern, two other members of the *A. tomentosa* group, clearly differ by lacking flavaglines. Instead, sesamin-type lignans were isolated from the former (Bachratá 2008), and sulfur-containing bisamides and related amide-esters from the latter species (Greger et al. 2008).

In spite of the fragmentary reports on flavaglines of the section *Amoora*, some general chemical trends became apparent. In contrast to section *Aglaia* the benzofurans were shown to be devoid of nitrogen and were mainly characterized by a 3′,4′-methylenedioxy substitution of ring B. The pronounced formation of 4′-demethoxy- 3′,4′-methylenedioxyaglafoline (**18**) represents a characteristic chemical feature reported so far for *A. spectabilis* (Miq.) Jain & Bennet (Schneider et al. 2000), *A. dasyclada* Miq. (Chaidir et al. 2001), *A. erythrosperma* C. M. Pannell (Phongmaykin et al. 2011) and *A. meridionalis* C. M. Pannell (Brem 2002), and is shown to be frequently accompanied by 4′-demethoxy- 3′,4′-methylenedioxyrocaglaol (**61**)**.** Moreover, another chemical feature of the section *Amoora* appears to be the occurrence of the rare rocagloic acid (**1**) in *A. cucullata* (Roxb.) Pellegrin (Chumkaew et al. 2006) and *A. rubiginosa* (Hiern) C. M. Pannell (Rivero-Cruz et al. 2004), and the bisamide aglairubine (Table 1B). With regard to the confined distribution of this chemical profile, the coexisting 3′,4′-methylenedioxy derivatives of aglafoline (**18**, **19**) and rocaglaol (**61**, **63**) in *A. elliptica* Blume of the section *Aglaia* (Cui et al. 1997) was surprising and needs a re-identification of the plants. Even more, as different nitrogen-containing rocaglamide derivatives (**25**, **38**, **75**, **76**, **77**) were published for another collection of *A. elliptica* (Nugroho et al. 1997b), and many further accessions from Thailand, identified by C. M. Pannell, were uniformly characterized by a lack of flavaglines. In this case, the absence of flavaglines was also confirmed by parallel bioassays of crude extracts, where no insect toxicity was determined (Brem 2002). On the other hand it should be noted, that the “*Amoora*-profile” with 3′,4′-methylenedioxy substitution in ring B (**18**, **19**, **21**, **61**, **62**) was also reported for an accession of *A. perviridis* Hiern of the section *Aglaia*. However, the profile differed by an additional formation of the nitrogen-containing didesmethylrocaglamide (**38**), and of rocaglaol (**54**) and aglafoline derivatives (**4**, **5**, **11**). It might be of chemosystematic relevance, that here the new benzopyrans perviridisins A and B (**106**, **107**) were isolated, whose bisamide moieties are characterized by aglairubine (Pan et al. 2013). In a re-investigation of the roots of *A. perviridis*, originating from the same locality in Vietnam, the highly bioactive silvestrols **14** and **15** were isolated along with a new type of closely related derivatives (**41**–**46**). However, in contrast to the previous study no indication was given for coexisting 3′,4′-methylenedioxy substituted benzofuran derivatives (Agarwal et al. 2019). The formation of silvestrol (**14**) in *A. perviridis* was confirmed in another collection from south China, where it was isolated together with a series of benzopyrans (**116**–**118**, **121**, **122**), whose bisamide moieties are characterized by benzoic and phenylacetic acid moieties (An et al. 2016). Regarding the restricted occurrence of silvestrol (**14**), only known so far from the four species *A. foveolata* C. M. Pannell (Hwang et al. 2004), *A. leptantha* Miq. (Meurer-Grimes et al. 2004), *A. perviridis* Hiern (An et al. 2016; Agarwal et al. 2019), and *A. stellatopilosa* C. M. Pannell (Othman et al. 2016), this rare structural modification of flavaglines can be expected as another chemical feature to characterize a group of related species.^3^

## Footnotes

1 A list of corresponding voucher specimens and GenBank accession numbers is presented in Muellner et al. (2009)

2 the name aglafoline (**4**) was published together with the correct chemical structure by Ko et al. (1992), whereas methyl rocaglate by Ishibashi et al. in the year 1993, so the compound name from Ko et al. takes priority.

3 After finalizing the present review, a parallel report appeared on different classes of compounds of *Aglaia* including flavaglines (Harneti and Supratman (2021)).
